# Phase Ib/II Study of Pamiparib Plus Radiation Therapy and/or Temozolomide in Adult Patients with Treatment-Naïve or Recurrent/Refractory Glioblastoma

**DOI:** 10.3390/curroncol32100541

**Published:** 2025-09-27

**Authors:** Anna F. Piotrowski, Kent Shih, Pierre Giglio, Howard Colman, Patrick Y. Wen, Jian Li Campian, Nicholas Butowski, Timothy Cloughesy, Zhaoyin Zhu, Vitaliy Gisin, Michael Badruddoja

**Affiliations:** 1 Department of Neurology, Memorial Sloan Kettering Cancer Center, New York, NY 10065, USA; piotrowa@mskcc.org; 2 Medical Oncology, Tennessee Oncology, PLLC, Nashville, TN 37203, USA; kshih@tnonc.com; 3 Department of Neurology, The Ohio State University, Columbus, OH 43210, USA; pierre.giglio@osumc.edu; 4 Department of Neurosurgery, Huntsman Cancer Institute, University of Utah, Salt Lake City, UT 84112, USA; howard.colman@hci.utah.edu; 5 Department of Neurology, Dana-Farber Cancer Institute, Boston, MA 02215, USA; patrick_wen@dfci.harvard.edu; 6 Division of Oncology, Washington University, St Louis, MO 63110, USA; campian.jian@mayo.edu; 7 Department of Neuro-Oncology, University of California San Francisco, San Francisco, CA 94143, USA; nicholas.butowski@ucsf.edu; 8 Department of Neurology, Bowyer Oncology Center, University of California, Los Angeles, CA 90095, USA; tcloughesy@mednet.ucla.edu; 9 Clinical Development, BeOne Medicines, Ltd., Ridgefield Park, NJ 07660, USA; zhaoyin.zhu@beonemed.com; 10 Clinical Development, BeOne Medicines, Ltd., Emeryville, CA 94608, USA; vgisin@gmail.com; 11 Department of Neurology, Center for Neurosciences, Tucson, AZ 85718, USA

**Keywords:** clinical trial, PARP inhibitor, pamiparib, phase II, glioblastoma

## Abstract

**Simple Summary:**

Glioblastoma is an aggressive brain cancer with poor survival rates, particularly in patients whose tumors lack a specific genetic marker (chemically modified or methylated O-6-methylguanine-DNA methyltransferase [*MGMT*]). This study tested pamiparib, a drug that blocks DNA repair, combined with radiation therapy and/or low-dose chemotherapy with temozolomide in 116 patients with newly diagnosed or recurrent glioblastoma. Patients received one of three treatments: pamiparib with radiation, pamiparib with both radiation and temozolomide, or pamiparib with temozolomide for recurrent tumors. The study found that these combinations were well-tolerated regardless of whether the glioblastoma was newly diagnosed or recurrent. The most common side effects were tiredness and nausea. In newly diagnosed patients, 67.9% had their tumor stop growing with 11.3% showing tumor shrinkage, and median overall survival (length of time patients are alive after the start of treatment) of 12.8 months. In patients with recurrent glioblastoma, 40.9% had their tumor stop growing with 13.6% showing tumor shrinkage, and median overall survival of 7.3 months. These results suggest pamiparib combinations may offer a promising treatment approach for patients with glioblastoma, particularly those newly diagnosed with unmethylated *MGMT* tumors who typically have fewer effective treatment options.

**Abstract:**

Pamiparib, a small-molecule poly (ADP-ribose) polymerase (PARP) 1/2 inhibitor, demonstrates strong PARP-DNA complex trapping, antitumor activity, and blood–brain barrier penetration. This phase Ib/II dose-escalation study (NCT03150862) investigated pamiparib’s tolerability/safety and efficacy when combined with radiotherapy and/or low-dose temozolomide (TMZ) in patients with treatment-naïve (Arms A and B) and recurrent/refractory (Arm C) glioblastoma. The recommended phase II dose for Arm A was pamiparib 60 mg twice daily (BID) for 6 weeks with 6–7 weeks radiotherapy; the recommended dose for Arm C was pamiparib 60 mg BID plus 60 mg TMZ (days 1–7; 28-day cycle). The Arm B escalation cohort completed enrollment; the expansion cohort was not opened. Grade ≥3 treatment-emergent adverse events (TEAEs)/serious TEAEs were observed in 55.0%/36.7% (Arm A), 44.4%/22.2% (Arm B), and 66.0%/38.3% (Arm C) of patients. Disease control and objective response rates were 67.9% and 11.3%, respectively, for treatment-naïve patients in the dose-escalation and -expansion studies, and 40.9% and 13.6%, respectively, for recurrent/refractory patients. Median overall survival for treatment-naïve *MGMT* unmethylated patients was 12.8 months and 7.3 months for recurrent/refractory *MGMT* methylated and unmethylated patients. Pamiparib with radiotherapy and/or low-dose TMZ was tolerable for treatment-naïve or recurrent/refractory glioblastoma. Treatment-emergent cytopenia was manageable and reversible with dose reductions/interruptions. Combination regimens demonstrated antitumor activity.

## 1. Introduction

Glioblastoma is a highly aggressive cancer, representing almost half (49%) of all brain and central nervous system malignancies, and it has a poor prognosis, with a median overall survival (OS) of approximately 15 months [1]. Studies have consistently demonstrated a higher incidence of glioblastoma in males compared to females [1]. The 5-year survival rate is 6.8%, which decreases substantially with older age at diagnosis [2,3]. Prognosis is worse in patients without methylation of the promoter region of the O-6-methylguanine-DNA methyltransferase (*MGMT*) gene [2,4].

First-line treatment of glioblastoma consists of maximal safe surgical resection, often followed by radiotherapy (RT) and chemotherapy [5,6,7,8,9]. A pivotal study in 2005 established temozolomide (TMZ) as the standard of care for newly diagnosed glioblastoma, showing an OS benefit [10]. However, clinical benefit from TMZ is largely limited to patients with a methylated *MGMT* promoter [8], which is a positive predictive and prognostic biomarker.

The combination of TMZ with poly (ADP-ribose) polymerase (PARP) 1/2 inhibitors has been investigated in various cancers [11,12,13,14,15,16]. PARP inhibitors interfere with DNA repair by inhibiting PARP enzymes and trapping PARP-DNA complexes at the DNA damage site [17,18,19,20,21,22]. PARP physically binds with poly (ADP) ribosylates *MGMT* in mice when combined with TMZ, providing a rationale for combining PARP inhibitors with TMZ for glioblastoma [23]. PARP inhibition can restore TMZ chemosensitivity in mismatch repair-deficient glioblastoma models [24]. However, myelosuppression has been reported as a dose-limiting toxicity (DLT) in studies evaluating TMZ with PARP inhibitors (olaparib, rucaparib, and veliparib) [25,26,27,28]. PARP inhibitors can also enhance the effects of RT by increasing the number of unrepaired DNA double-strand breaks. This replication-dependent mechanism protects healthy tissues while sensitizing highly proliferative tumors, thereby providing a rationale for combining PARP inhibitors with RT [29,30,31].

Pamiparib is a clinical-stage, small-molecule PARP 1/2 inhibitor that has demonstrated formation of strong PARP-DNA complexes, potent antitumor activity, and blood–brain barrier penetration in preclinical studies [22]. These studies demonstrated that pamiparib has high bioavailability and sustained activity, and is more potent than other drugs in this class in vivo xenograft models. Furthermore, pamiparib potentiated the antiproliferative effects of TMZ in glioblastoma cells, exerted antitumor synergism with TMZ in an animal model, and exhibited strong blood–brain barrier penetration [22]. Unlike other PARP inhibitors, pamiparib is not a substrate of P-glycoprotein; therefore, delivery across the blood–brain barrier is less restricted [32,33]. In early-phase clinical studies, single-agent pamiparib demonstrated antitumor activity and was well tolerated in patients with advanced solid tumors [32,33,34]. Taken together, these studies support the hypothesis that patients with newly diagnosed or recurrent/refractory glioblastoma may benefit from the addition of pamiparib to standard RT, with or without low-dose TMZ. Here, we report the safety and efficacy results of a phase Ib/II dose-escalation study of pamiparib in combination with RT and/or TMZ in patients with treatment-naïve, *MGMT*-unmethylated glioblastoma and recurrent/refractory glioblastoma with unmethylated or methylated *MGMT* (NCT03150862 and EudraCT 2017-001554-33).

## 2. Materials and Methods

### 2.1. Study Design

This open-label, multicenter, multiple-dose, dose-escalation phase Ib/II study evaluated the safety, tolerability, and efficacy of pamiparib in combination with RT and/or low-dose TMZ in patients with treatment-naïve or recurrent/refractory glioblastoma (Clinicaltrials.gov, NCT03150862, registration date: 8 May 2017). The trial was also registered on EudraCT (2017-001554-33). The protocol (BGB-290-104) was approved by the relevant institutional review board/independent ethics committee for each study site (Supplementary Table S1). This study was conducted in accordance with the International Council for Harmonisation Good Clinical Practice Guideline, principles of the Declaration of Helsinki, and local laws and regulations.

**Figure 1:**
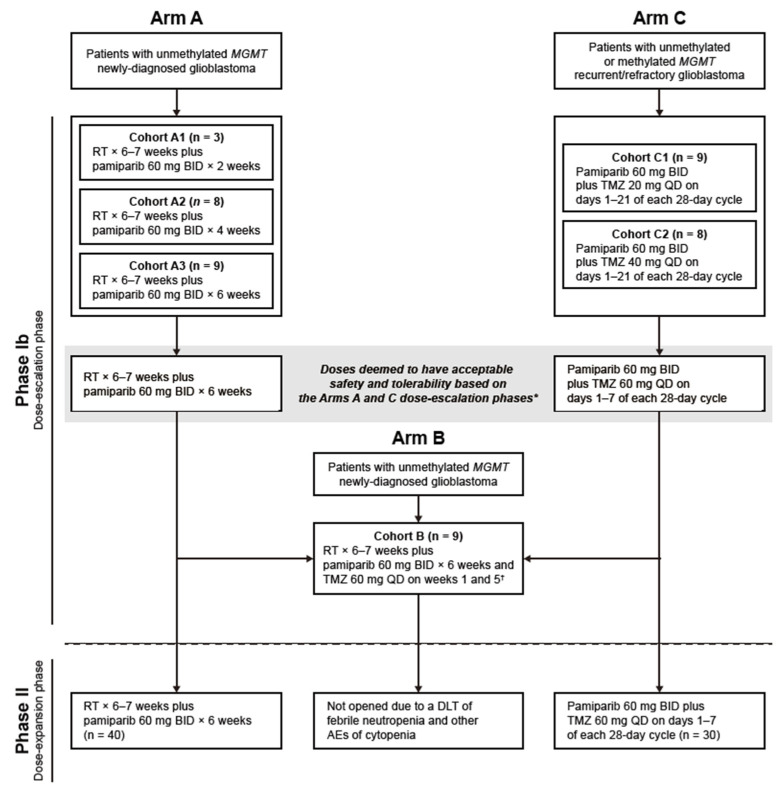
Study design. * In addition to data from the Arm C dose-escalation phase, safety data from the BGB-290-103 study, which assessed pamiparib plus TMZ in patients with advanced solid tumors, was considered to arrive at the dose of TMZ investigated in the Arm C dose-expansion phase; ^†^ The doses of pamiparib and TMZ investigated in the Arm B dose-escalation phase were chosen based on those selected for investigation in the Arm A and Arm C dose-expansion phases, respectively. Abbreviations: AE, adverse event; BID, twice daily; DLT, dose-limiting toxicity; QD, once daily; RT, radiotherapy; TMZ, temozolomide.

The study initially enrolled two cohorts: Arm A for newly diagnosed patients with unmethylated glioblastoma receiving pamiparib in combination with RT and Arm C for patients with recurrent/refractory glioblastoma (with unmethylated or methylated *MGMT*) receiving pamiparib plus TMZ (Figure 1). Based on the safety findings in Arm A, patients with newly diagnosed unmethylated glioblastoma were later enrolled into Arm B to receive pamiparib combined with both RT and TMZ (TMZ initiated at a dose determined in Arm C).

### 2.2. Participants

Patients were recruited from 20 sites in the USA, the Netherlands, and Switzerland, and all patients provided written informed consent. Eligible patients, aged ≥18 years, had histologically confirmed glioblastoma (World Health Organization [WHO] Grade IV, with supratentorial component) [35], Eastern Cooperative Oncology Group performance status (ECOG PS) of 0 or 1, adequate hematologic and end-organ function, ability to undergo serial magnetic resonance imaging (MRI) scans, and had undergone a brain MRI scan within 14 days prior to starting study treatment. All patients requiring glucocorticoids were on a daily dose equivalent to dexamethasone 8 mg or less that had been stable for ≥7 days prior to the MRI. All patients agreed to provide tumor tissue for exploratory biomarker analyses and documentation of *MGMT* promoter status (unmethylated/methylated). Full study inclusion/exclusion criteria are listed in the Supplementary Methods.

### 2.3. Study Cohorts and Treatments

In the phase Ib dose-escalation portion of the study, patients in Arm A received pamiparib (60 mg twice daily [BID]; 20 mg or 60 mg capsules depending on dose level and availability) administered continuously at increasing exposures of 2, 4, and 6 weeks in combination with RT for each cohort (total target dose: 58–64 Gy) administered for 6–7 weeks. Dependent on the safety profile of Arm A, patients in Arm B received pamiparib (dosage as determined from Arm A) in combination with RT administered for 6–7 weeks and low-dose TMZ (dosage as determined from Arm C). Patients received no further RT after these regimens were completed. Following completion of RT, at the discretion of the investigator and after discussion with the medical monitor, patients in the Arm A escalation and expansion phases and the Arm B escalation phase could receive maintenance treatment of pamiparib plus low-dose TMZ (at an equal or lower dose level than that determined to have acceptable safety and tolerability in Arm C). Maintenance treatment began after the 4-week rest period (+7 days) to allow for recovery from RT treatment. Patients continued maintenance treatment until progressive disease (PD), adverse events (AEs), death, voluntary withdrawal of consent, initiation of other anticancer therapy, or investigator’s decision. The Arm B dose-expansion cohort was not opened. In the Arm C dose-escalation cohort, patients received pamiparib (60 mg BID) administered continuously in combination with increasing doses of TMZ (20 mg and 40 mg) administered on days 1–21 of each 28-day cycle. In the phase II dose-expansion cohort, up to 60 patients could be enrolled in each expansion cohort at a dose level below or equal to the maximum tolerated dose for that arm. Patients continued receiving pamiparib in combination with RT and/or low-dose TMZ until PD, unacceptable toxicity, death, withdrawal of consent, loss to follow-up, or study termination by the sponsor. Patients were prohibited from receiving any other anticancer therapy, including surgery and RT, other than that specified in the protocol. Patients in Arms A and B who completed all study treatments per protocol and did not continue on maintenance treatment were scheduled to have their end-of-treatment (EOT) visit 28 days after completing RT. For all other patients, an EOT visit was required within 7 days of stopping all study treatment.

### 2.4. Endpoints

#### 2.4.1. Primary and Secondary Endpoints: Phase Ib Study

For the phase Ib study, the primary endpoint was to assess safety and tolerability by monitoring non-serious and serious AEs, relevant physical examinations, electrocardiograms, and laboratory assessments as needed. Additional coprimary endpoints were included to identify DLTs, determine the number of cycles (Arm C only) and the dose intensity of each component of the treatment regimens, and identify changes in vital signs and clinical laboratory test results during and following study treatment. Secondary endpoints included a preliminary assessment of pamiparib efficacy in combination with RT and/or TMZ.

#### 2.4.2. Primary and Secondary Endpoints: Phase II Study

For the phase II study, the primary endpoint in Arms A and B was modified disease control rate (DCR) per Response Assessment in Neuro-Oncology (RANO) criteria [36] at the EOT visit. The primary endpoint in Arm C was objective response rate (ORR) per RANO criteria. Key secondary endpoints in Arms A and B included ORR and clinical benefit rate per RANO criteria. In Arm C, key secondary endpoints included DCR and clinical benefit ratio per RANO criteria. Other secondary endpoints in all treatment arms included duration of response (DoR), progression-free survival (PFS), OS, safety and tolerability, dose intensity of each component of the treatment regimens, and changes in vital signs and clinical laboratory test results during and following study treatment.

### 2.5. Assessments

Antitumor activity was assessed by MRI. Following tumor assessment at screening (within 14 days of day 1), tumor assessments occurred every 4 weeks ± 7 days (for Arms A and B, the post-screening assessments commenced 4 weeks ± 7 days after completion of RT) and as part of the EOT visit. Patients without PD at EOT were followed with MRI scans every 4 or 8 weeks ± 7 days, at the discretion of the investigator, until PD or study discontinuation, whichever was earlier.

Over the course of the study, and up to 30 days after their final treatment dose or initiation of their new anticancer therapy (which ever occurred first), patients had assessments for safety and tolerability. All AEs were systematically classified using the Medical Dictionary for Regulatory Activities (version 23.0) and their severity was evaluated as per the National Cancer Institute Common Terminology Criteria for Adverse Events (version 4.03). Adverse events were considered treatment-emergent (TEAEs) if they had an onset date on or after the first study treatment administration or showed worsening in severity from baseline (pretreatment) up to 30 days following permanent study treatment discontinuation or initiation of new anticancer therapy, whichever occurred first.

### 2.6. Statistical Analyses

The safety analysis set included all patients who received ≥1 dose of pamiparib, RT, and/or low-dose TMZ, and this group was used to evaluate PFS, OS, and safety outcomes. The efficacy analysis set consisted of the patients in the safety population who had completed tumor assessments at both baseline and EOT (for Arms A and B) or had measurable disease at baseline and at least one follow-up tumor assessment (for Arm C), excluding those who discontinued treatment or the study early due to PD or death prior to tumor evaluation. Tumor response endpoints were evaluated using the efficacy analysis set. The DLT analysis set comprised the patients from the dose-escalation cohorts in the safety analysis set who received at least 42 Gy of RT (Arms A and B), at least 70% of planned pamiparib treatment in all three arms, and at least 70% of scheduled low-dose TMZ (Arms B and C) during the DLT assessment period. Any patient experiencing a DLT event was considered evaluable for this analysis regardless of their study treatment intensity.

The study planned to enroll approximately 300 patients (60 in phase Ib; 240 in phase II); however, the sample size was reduced during the study because the expansion cohorts were considered too large for this non-randomized study with respect to statistical hypotheses and key study design parameters. Furthermore, the Arm B expansion cohort was not opened. In phase II of the study, the initial sample size was calculated based on modified DCRs in Arms A and B, and ORR for each Arm C expansion cohort. With an assumed modified DCR of 60% in Arms A and B compared with 40% in the historical control, the study was designed to have approximately 82% statistical power to detect a 20% difference in modified DCR using a one-sided alpha of 0.025 and enrolling 60 patients in each expansion cohort. Following the reduction to an actual sample size of 40 patients, and still assuming a modified DCR of 60%, the analysis determined that a minimum observed modified DCR of 53% (21 of 40 patients) would be needed to achieve an 80% exact confidence interval (CI) excluding the historical control rate of 40%. An assumption of 25% ORR in Arm C, compared with 10% in the historical control, provided the study a statistical power of approximately 85% to detect a 15% ORR difference using a one-sided alpha of 0.025 and enrolling 60 patients in each expansion cohort. Similarly, after the actual sample size was reduced to 30 patients and maintaining the assumption of an observed ORR of 25%, the analysis indicated that a minimum observed ORR of 20% (six of 30 patients) would be required to achieve an 80% exact CI effectively excluding the historical control rate of 10%.

Assessment of precision of rate estimates included construction of two-sided binomial exact 95% CIs of modified DCR and ORR. The study also presented the number and percentage of patients achieving best overall response categories (complete response, partial response, stable disease, and PD). The Kaplan–Meier method was used to estimate time-to-event variables PFS, OS, and DoR, and these estimates were plotted over time. Median PFS, DoR, and OS in each arm were estimated if possible and reported along with their two-sided 95% CIs calculated using the Brookmeyer and Crowley method. The Kaplan–Meier method was also used to estimate 6- and 12-month PFS (defined as the proportion of patients in the safety analysis set remaining alive and progression-free at these time points) and OS (defined as the proportion of patients in the safety analysis set remaining alive at these time points), with the corresponding 95% CIs determined using Greenwood’s formula.

## 3. Results

### 3.1. Patient Characteristics

The median age of patients with newly diagnosed glioblastoma enrolled in Arms A and B was 61.0 years (range: 31–79), with 65.2% aged <65 years. Most patients were male (65.2%), White (92.8%), had an ECOG PS of 1 (65.2%), and had undergone complete or partial resection at initial glioblastoma diagnosis (49.3% and 40.6%, respectively) (Table 1). Median study follow-up was 12.0 months (range: 0–22).

Among patients with recurrent/refractory glioblastoma (Arm C), the median age was 55.0 years (range: 24–87), with 83.0% aged <65 years. Most patients were male (68.1%), White (89.4%), and had an ECOG PS of 1 (68.1%). Median time from initial diagnosis to study entry was 14.2 months (Table 2). Median study follow-up was 7.1 months (range: 1–25).

### 3.2. Treatment Exposure

Overall, median duration of pamiparib treatment in Arms A and B was 6.1 weeks (range: 0.7–11.1), regardless of whether it was administered with RT alone or RT with low-dose TMZ. Median average pamiparib dose intensity per patient was 115.8 mg/day. During the maintenance phase for Arms A and B, median treatment duration was 13.6 weeks (range: 0.4–45.6). In Arm C, overall median treatment duration was 7.3 weeks (range: 0.4–107.8); median average pamiparib dose intensity per patient was 116.5 mg/day.

### 3.3. Safety and Tolerability

Among 46 patients in the dose-escalation phase, nine DLTs occurred in seven patients. In Arm A, two patients in the pamiparib 60 mg BID for 6 weeks with RT cohort reported DLTs, including one patient with vertigo and chills, and one with fatigue. Pamiparib 60 mg BID for 6 weeks with RT was deemed to have acceptable safety and tolerability and was recommended for further assessment in the expansion phase. In Arm C, DLTs were reported in three patients in the pamiparib 60 mg BID with 40 mg TMZ once daily (QD) cohort, including one patient with nausea and vomiting, one with neutropenia, and one with neutrophil count decreased. The dosing schedule investigated in the Arm C expansion phase (pamiparib 60 mg BID with 60 mg of TMZ QD on days 1–7 of each 28-day cycle) was determined based on safety data from the Arm C dose-escalation phase and the BGB-290-103 study (NCT03150810), which assessed the same treatment combination in patients with advanced solid tumors [37]. The Arm B dose-escalation phase was opened to investigate RT in combination with the doses of pamiparib and TMZ selected in the escalation phases of Arms A and C, respectively (pamiparib 60 mg BID for 6 weeks and 60 mg TMZ QD during weeks 1 and 5). Due to a DLT of febrile neutropenia and other AEs of cytopenia, Arm B was not further escalated or expanded thereafter.

Safety and tolerability for patients with newly diagnosed glioblastoma (Arms A and B) are summarized in Table 3 and Supplementary Tables S2, S3, S5, S6, S8 and S9. All 60 patients (100%) in Arm A had one or more TEAE during the dose-escalation and -expansion phases, with 33 (55.0%) experiencing one or more TEAE that was grade ≥3. Fatigue (66.7%) and nausea (63.3%) were the most common TEAEs. Few grade ≥3 TEAEs or serious TEAEs were assessed as related to pamiparib treatment (five patients [8.3%] and three patients [5.0%], respectively). In Arm B, all nine patients (100%) experienced one or more TEAE, with four (44.4%) experiencing one or more TEAE that was grade ≥3. Nausea (77.8%) and fatigue (66.7%) were the most common TEAEs. TEAEs assessed as related to pamiparib were experienced by three patients (33.3%), and TEAEs related to TMZ were experienced by five patients (55.6%). One patient (11.1%) experienced a grade ≥3 TEAE assessed as related to pamiparib; no patients reported serious TEAEs related to pamiparib or TMZ. Two patients in Arm A and one patient in Arm B had grade 4 hematologic toxicities (leukocyte and neutrophil counts decreased).

Safety and tolerability for patients with recurrent/refractory glioblastoma (Arm C) are summarized in Supplementary Tables S4, S7 and S10. Forty-six patients (97.9%) experienced a TEAE during any study phase, with 31 (66.0%) experiencing a TEAE that was grade ≥3. The most common TEAEs were fatigue (48.9%) and nausea (46.8%). TEAEs assessed as related to pamiparib were experienced by 11 patients (23.4%), and TEAEs assessed as related to TMZ treatment were experienced by 16 patients (34.0%). The most common (≥5% of patients) grade ≥3 treatment-related TEAEs were lymphocyte count decreased, fatigue, and anemia (five patients each [10.6%]); neutrophil count decreased, white blood cell count decreased, platelet count decreased, and neutropenia (four patients each [8.5%]); and thrombocytopenia, confusional state, muscular weakness, and nausea (three patients each [6.4%]). Six patients in Arm C had grade 4 hematologic toxicities (neutrophil, lymphocyte, leukocyte, and platelet counts decreased).

### 3.4. Efficacy

Efficacy endpoints for Arms A and B are summarized in Table 4, and for Arm C in Table 5. In patients with newly diagnosed glioblastoma in Arms A and B, the primary endpoint of modified DCR per RANO criteria at the EOT visit (unconfirmed) was 67.9% (95% CI: 53.7–80.1) and the unconfirmed ORR was 11.3% (95% CI: 4.3–23.0) in the efficacy analysis set. Median PFS and OS in the safety analysis set were 4.4 months (95% CI: 3.4–6.1) and 12.8 months (95% CI: 10.3–14.2), respectively. The estimated 6-month PFS event-free rate was 39.5% (95% CI: 25.9–52.7) (Table 4).

For patients with recurrent/refractory glioblastoma in Arm C, the primary endpoint of ORR per RANO criteria (unconfirmed) was 13.6% (95% CI: 5.2–27.4) in the efficacy analysis set. Median PFS and OS in the safety analysis set were 1.9 months (95% CI: 1.7–2.3) and 7.3 months (95% CI: 6.2–9.8), respectively. The estimated 6-month PFS event-free rate was 17.2% (95% CI: 7.6–30.1) (Table 5).

## 4. Discussion

The combination of pamiparib over the 6-week treatment period with standard-of-care RT in newly diagnosed glioblastoma (Arm A) was tolerable. The safety profile was consistent with other oral PARP inhibitors plus RT [38], despite a slightly older study population (median: 60.0–65.5 years) [10,39]. Pamiparib (60 mg BID) showed an acceptable safety profile over 6 weeks with a combination of RT and TMZ in patients with newly diagnosed glioblastoma. Efficacy outcomes for pamiparib plus RT, with or without TMZ, demonstrated encouraging antitumor activity. It should be noted that both Arms A and B in this study included only patients with unmethylated *MGMT* promoter status, who typically have poorer OS [40]. A recent meta-analysis reported a pooled median OS of 14.1 months in patients with an unmethylated *MGMT* promoter treated with RT plus TMZ [40], similar to the median OS (14.2 months) observed in Arm B of the current study.

In Arm C (recurrent/refractory glioblastoma), pamiparib combined with TMZ showed acceptable safety and tolerability, with the TEAEs of cytopenia being manageable and reversible with dose reductions and/or interruptions. However, the ORR (unconfirmed) of 13.6% with pamiparib plus TMZ showed no improvement over the historical control of 14.0% with TMZ alone, which served as the protocol benchmark [41]. A 2023 meta-analysis reported a lower ORR of 7.6% for TMZ in recurrent glioblastoma [42], making the ORR with pamiparib plus TMZ in Arm C comparatively favorable. The median OS of 7.3 months in Arm C showed no improvement over that previously reported with bevacizumab (9.2 months) and lomustine (7.1 months) in patients with recurrent glioblastoma [43,44]. The limited efficacy in Arm C may be partially explained by the length between initial diagnosis and study entry (14.2 months); the majority of patients had unmethylated *MGMT* promoter, and there was a high proportion of patients previously treated with TMZ (97.9%) or at second relapse (14.9%), indicating a heavily pretreated population. Therefore, effective treatments are still needed for patients with recurrent/refractory glioblastoma.

Future research may reveal additional treatment candidates for combination with pamiparib. Various glioma studies are investigating PARP inhibitors with RT or tumor-treating fields, chemotherapy, or antiangiogenics (e.g., TMZ, bevacizumab), and immunotherapeutic agents (e.g., durvalumab [an anti-programmed death-ligand 1]) [45]. The recent OLA-TMZ-RTE-01 trial demonstrated promising outcomes with intermittent olaparib dosing in combination with standard radiochemotherapy. These findings complement our observations with pamiparib and support further investigation of optimized dosing schedules [31]. The PARADIGM-2 trial, which consisted of two parallel phase I studies of olaparib and RT or olaparib and RT plus TMZ in patients with newly diagnosed glioblastoma, used a biomarker-driven approach where *MGMT* status guides PARP inhibitor dosing intensity with RT, recognizing that patients with unmethylated *MGMT* promoter derive minimal benefit with TMZ [30]. While further translational work is required to identify biomarkers of treatment response to inform the optimal use of PARP inhibitors in glioma, this represents a promising area of neuro-oncology research [45].

As is typical in phase Ib/II dose-escalation and dose-expansion studies, the results should be interpreted cautiously due to low patient numbers in some cohorts/arms. The study did not include sex-specific analyses for safety and efficacy outcomes. However, the higher percentage of male participants aligns with the well-established higher incidence of glioblastoma in males [1]. Due to the reduced sample size in the Arm C expansion cohort, the analysis did not separate patients by *MGMT* methylation status. Additionally, the present study did not determine the isocitrate dehydrogenase (*IDH*) mutational status of prospective patients. According to the most recent WHO Classification of Tumors of the central nervous system, glioblastomas are classified by presence of wildtype *IDH* status [46]. As the study completed enrollment prior to 2021, previous WHO classification guidelines were followed [35] and *IDH* mutational status was not evaluated. Previously, multivariate analyses revealed that patients with *IDH1*-mutant glioblastoma, now classified as grade 4 astrocytoma, and *MGMT* promoter methylation exhibited improved survival compared with patients having one or neither of these molecular alterations [46,47]. Given the majority of patients with unmethylated *MGMT* likely had wildtype *IDH*, the patients in this study cohort were expected to have worse outcomes. Although pamiparib previously demonstrated antitumor activity in cell lines with breast cancer genes 1/2 (*BRCA1/2*) mutations or other homologous recombination deficiencies [22], its effect on homologous recombination deficiency was not assessed in the current study. This is an area of interest for future studies, as current data suggest that pamiparib may be particularly suitable for patients with tumors harboring these mutations [22].

## 5. Conclusions

In conclusion, pamiparib with RT and/or low-dose TMZ was tolerable in patients with newly diagnosed or recurrent/refractory glioblastoma, with the TEAEs of cytopenia being manageable and reversible with dose reductions and/or interruptions. The combination regimens showed antitumor activity in treatment-naïve and recurrent/refractory glioblastoma, but larger studies are needed to expand on these initial findings.

## Figures and Tables

**Table 1:** Demographics and baseline characteristics of patients with newly diagnosed glioblastoma (Arm A and Arm B; safety analysis set).

Characteristic/ Demographic	Arm A: Dose-Escalation Phase			Arm A: Dose-Expansion Phase	Arm B: Dose-Escalation	Total Newly Diagnosed Glioblastoma (N = 69)
	Pamiparib 2 Weeks + RT (N = 3)	Pamiparib 4 Weeks + RT (N = 8)	Pamiparib 6 Weeks + RT (N = 9)	Pamiparib 6 Weeks + RT (N = 40)	Pamiparib 6 Weeks + RT + TMZ 60 mg Weeks 1 and 5 (N = 9)	
Median age, years (range)	64.0 (50–65)	65.5 (43–71)	60.0 (42–68)	58.0 (31–79)	62.0 (45–77)	61.0 (31–79)
Age group, n (%)						
<65 years	2 (66.7)	3 (37.5)	7 (77.8)	28 (70.0)	5 (55.6)	45 (65.2)
≥65 years	1 (33.3)	5 (62.5)	2 (22.2)	12 (30.0)	4 (44.4)	24 (34.8)
Sex, n (%)						
Female	0 (0.0)	1 (12.5)	2 (22.2)	17 (42.5)	4 (44.4)	24 (34.8)
Race, n (%)						
White	3 (100.0)	7 (87.5)	7 (77.8)	38 (95.0)	9 (100.0)	64 (92.8)
ECOG performance status, n (%)						
0	1 (33.3)	2 (25.0)	3 (33.3)	13 (32.5)	5 (55.6)	24 (34.8)
1	2 (66.7)	6 (75.0)	6 (66.7)	27 (67.5)	4 (44.4)	45 (65.2)
Median time from initial diagnosis to study entry, weeks (range)	4.1 (3–5)	2.7 (2–6)	3.7 (2–4)	3.4 (1–8)	3.1 (2–4)	3.3 (1–8)
Median time since initial surgical resection, weeks (range)	4.1 (3–5)	2.9 (2–6)	3.8 (2–4)	3.4 (1–8)	3.0 (1–4)	3.3 (1–8)
Surgical status for initial diagnosis of glioblastoma, n (%)						
Biopsy	1 (33.3)	2 (25.0)	1 (11.1)	2 (5.0)	0 (0.0)	6 (8.7)
Complete resection	2 (66.7)	3 (37.5)	3 (33.3)	21 (52.5)	5 (55.6)	34 (49.3)
Partial resection	0 (0.0)	3 (37.5)	5 (55.6)	16 (40.0)	4 (44.4)	28 (40.6)
Other (left temporal mass debulking)	0 (0.0)	0 (0.0)	0 (0.0)	1 (2.5)	0 (0.0)	1 (1.4)
Baseline corticosteroid use, n (%)						
Yes	3 (100.0)	4 (50.0)	6 (66.7)	18 (45.0)	5 (55.6)	36 (52.2)

Data cutoff: 13 April 2021. Abbreviations: ECOG, Eastern Cooperative Oncology Group; RT, radiotherapy; TMZ, temozolomide.

**Table 2:** Demographics and baseline characteristics of patients with recurrent/refractory glioblastoma (Arm C; safety analysis set).

Characteristic/ Demographic	Arm C: Dose-Escalation Phase		Arm C: Dose-Expansion Phase	Total Recurrent/Refractory Glioblastoma (N = 47)
	Pamiparib + TMZ 20 mg Days 1–21 (N = 9)	Pamiparib + TMZ 40 mg Days 1–21 (N = 8)	Pamiparib + TMZ 60 mg Days 1–7 (N = 30)	
Median age, years (range)	53.0 (24–62)	53.5 (25–67)	58.0 (33–87)	55.0 (24–87)
Age group, n (%)				
<65 years	9 (100.0)	7 (87.5)	23 (76.7)	39 (83.0)
≥65 years	0 (0.0)	1 (12.5)	7 (23.3)	8 (17.0)
Sex, n (%)				
Female	1 (11.1)	4 (50.0)	10 (33.3)	15 (31.9)
Race, n (%)				
White	7 (77.8)	8 (100.0)	27 (90.0)	42 (89.4)
ECOG performance status, n (%)				
0	2 (22.2)	3 (37.5)	10 (33.3)	15 (31.9)
1	7 (77.8)	5 (62.5)	20 (66.7)	32 (68.1)
Median time from initial diagnosis to study entry, weeks (range)	15.1 (12–108)	24.2 (10–87)	10.9 (5–91)	14.2 (5–108)
Median time since initial surgical resection, weeks (range)	15.1 (11–108)	24.2 (10–87)	10.7 (4–91)	13.1 (4–108)
Surgical status for initial diagnosis of glioblastoma, n (%)				
Complete resection	6 (66.7)	7 (87.5)	15 (50.0)	28 (59.6)
Partial resection	3 (33.3)	1 (12.5)	15 (50.0)	19 (40.4)
Relapse status, n (%)				
First relapse	7 (77.8)	6 (75.0)	27 (90.0)	40 (85.1)
Second relapse	2 (22.2)	2 (25.0)	3 (10.0)	7 (14.9)
WHO grade at initial diagnosis of glioblastoma, n (%)				
Grade IV	9 (100.0)	6 (75.0)	29 (96.7)	44 (93.6)
*MGMT* promoter status, n (%)				
Methylated	1 (11.1)	3 (37.5)	12 (40.0)	16 (34.0)
Unmethylated	7 (77.8)	4 (50.0)	18 (60.0)	29 (61.7)
Prior use of TMZ, n (%)				
Yes	9 (100.0)	7 (87.5)	30 (100.0)	46 (97.9)
Baseline corticosteroid use, n (%)				
Yes	5 (55.6)	3 (37.5)	17 (56.7)	25 (53.2)

Data cutoff: 13 April 2021. Abbreviations: ECOG, Eastern Cooperative Oncology Group; *MGMT*, O-6-methylguanine-DNA methyltransferase; TMZ, temozolomide; WHO, World Health Organization.

**Table 3:** Overview of TEAEs in patients with newly diagnosed glioblastoma (Arms A and B) and recurrent/refractory glioblastoma (Arm C) (safety analysis set).

Events, N (%)	Newly Diagnosed Glioblastoma		Recurrent/Refractory Glioblastoma
	Arm A (N = 60) *	Arm B (N = 9) ^†^	Arm C (N = 47) ^‡^
Patients with ≥1 TEAE	60 (100.0)	9 (100.0)	46 (97.9)
TEAEs of grade ≥3	33 (55.0)	4 (44.4)	31 (66.0)
Treatment-emergent SAEs	22 (36.7)	2 (22.2)	18 (38.3)
TEAE leading to death	3 (5.0)	0 (0.0)	1 (2.1)
TEAE leading to treatment discontinuation of pamiparib only	0 (0.0)	0 (0.0)	0 (0.0)
TEAE leading to treatment discontinuation of RT only	1 (1.7)	0 (0.0)	–
TEAE leading to treatment discontinuation of TMZ only	0 (0.0)	0 (0.0)	0 (0.0)
TEAE leading to dose modification of pamiparib only	12 (20.0)	2 (22.2)	13 (27.7)
Leading to dose interruption	12 (20.0)	2 (22.2)	13 (27.7)
Leading to dose reduction	0 (0.0)	0 (0.0)	0 (0.0)
TEAE leading to dose modification of RT only	4 (6.7)	0 (0.0)	–
Leading to dose interruption	4 (6.7)	0 (0.0)	–
Leading to dose reduction	0 (0.0)	0 (0.0)	–
TEAE leading to dose modification of TMZ only	3 (5.0)	1 (11.1)	4 (8.5)
Leading to dose interruption	2 (3.3)	1 (11.1)	3 (6.4)
Leading to dose reduction	1 (1.7)	0 (0.0)	2 (4.3)
TEAE related to pamiparib only	38 (63.3)	3 (33.3)	11 (23.4)
TEAE related to RT only	39 (65.0)	6 (66.7)	–
TEAE related to TMZ only	5 (8.3)	5 (55.6)	16 (34.0)
TEAE related to pamiparib only grade ≥3	5 (8.3)	1 (11.1)	0 (0.0)
TEAE related to RT only grade ≥3	2 (3.3)	1 (11.1)	–
TEAE related to TMZ only grade ≥3	0 (0.0)	0 (0.0)	3 (6.4)
Serious TEAE related to pamiparib only	3 (5.0)	0 (0.0)	1 (2.1)
Serious TEAE related to RT only	1 (1.7)	0 (0.0)	–
Serious TEAE related to TMZ only	0 (0.0)	0 (0.0)	0 (0.0)
Treatment-related TEAEs leading to death	0 (0.0)	0 (0.0)	0 (0.0)
Treatment-related TEAE leading to treatment discontinuation of pamiparib only	0 (0.0)	0 (0.0)	0 (0.0)
Treatment-related TEAE leading to treatment discontinuation of RT only	0 (0.0)	0 (0.0)	–
Treatment-related TEAE leading to treatment discontinuation of TMZ only	0 (0.0)	0 (0.0)	0 (0.0)
Treatment-related TEAE leading to dose modification of pamiparib only	7 (11.7)	2 (22.2)	10 (21.3)
Leading to dose interruption	7 (11.7)	2 (22.2)	10 (21.3)
Leading to dose reduction	0 (0.0)	0 (0.0)	0 (0.0)
Treatment-related TEAE leading to dose modification of RT only	1 (1.7)	0 (0.0)	–
Leading to dose interruption	1 (1.7)	0 (0.0)	–
Leading to dose reduction	0 (0.0)	0 (0.0)	–
Treatment-related TEAE leading to dose modification of TMZ only	3 (5.0)	1 (11.1)	4 (8.5)
Leading to dose interruption	2 (3.3)	1 (11.1)	3 (6.4)
Leading to dose reduction	1 (1.7)	0 (0.0)	2 (4.3)

Data cutoff: 13 April 2021. Note: For each row category, a patient with two or more AEs in that category is counted only once. Treatment-related TEAEs are TEAEs that are considered by the investigator to be possibly or probably related to study drug or with missing assessment of the causal relationship. A treatment-related AE refers to an AE related to any of the study treatments of pamiparib, RT, or TMZ. AE grades are evaluated based on CTCAE (Version 4.03). * Includes all patients from the dose-escalation and dose-expansion Arm A cohorts. ^†^ Includes patients from the dose-escalation Arm B cohort (the Arm B dose-expansion cohort was not opened). ^‡^ Includes all patients from the dose-escalation and dose-expansion Arm C cohorts. Abbreviations: AE, adverse event; CTCAE, Common Terminology Criteria for Adverse Events; RT, radiotherapy; SAE, serious adverse event; TEAE, treatment-emergent adverse event; TMZ, temozolomide.

**Table 4:** Primary and secondary efficacy endpoints in newly diagnosed patients.

Endpoint	Arm A: Dose-Escalation Phase			Arm A: Dose-Expansion Phase	Arm B: Dose-Escalation	
	Pamiparib 2 Weeks + RT	Pamiparib 4 Weeks + RT	Pamiparib 6 Weeks + RT	Pamiparib 6 Weeks + RT	Pamiparib 6 Weeks + RT + TMZ 60 mg Weeks 1 and 5	Total Newly Diagnosed Glioblastoma
Disease Response Analyses (Unconfirmed; Efficacy Analysis Set *)						
N	3	6	7	32	5	53
Best response per RANO criteria, n (%)						
CR	0	0	0	0	0	0
PR	0	3 (50.0)	0	3 (9.4)	0	6 (11.3)
SD	2 (66.7)	3 (50.0)	3 (42.9)	19 (59.4)	4 (80.0)	31 (58.5)
PD	1 (33.3)	0	4 (57.1)	10 (31.3)	1 (20.0)	16 (30.2)
Modified disease control rate (CR, PR, or SD at EOT visit), n (%) [95% CI]	2 (66.7) [9.4–99.2]	6 (100.0) [54.1–100.0]	3 (42.9) [9.9–81.6]	21 (65.6) [46.8–81.4]	4 (80.0) [28.4–99.5]	36 (67.9) [53.7–80.1]
ORR, n (%) [95% CI]	0 [0.0–70.8]	3 (50.0) [11.8–88.2]	0 [0.0–41.0]	3 (9.4) [2.0–25.0]	0 [0.0–52.2]	6 (11.3) [4.3–23.0]
Clinical benefit rate (CR + PR + durable ^†^ SD), n (%) [95% CI]	0 [0.0–70.8]	4 (66.7) [22.3–95.7]	0 [0.0–41.0]	13 (40.6) [23.7–59.4]	2 (40.0) [5.3–85.3]	19 (35.8) [23.1–50.2]
DoR per RANO criteria						
Events, n (%)	0	1 (33.3)	0	3 (100.0)	0	4 (66.7)
Median, months (95% CI) ^‡^	NA	6.4 (NE–NE)	NA	3.8 (1.18–10.32)	NA	5.1 (1.18–10.32)
Survival analyses (safety analysis set ^§^)						
N	3	8	9	40	8	68
PFS						
Median, months (95% CI)	3.1 (2.8–3.3)	8.9 (3.8–11.6)	2.6 (2.1–NE)	4.4 (3.3–6.2)	5.8 (2.4–6.5)	4.4 (3.4–6.1)
Event-free rate, % (95% CI) ^#^						
6 months	0.0 (NE–NE)	66.7 (5.4–94.5)	NE (NE–NE)	42.7 (25.6–58.7)	38.1 (6.1–71.6)	39.5 (25.9–52.7)
12 months	0.0 (NE–NE)	0.0 (NE–NE)	NE (NE–NE)	6.6 (1.2–18.9)	0.0 (NE–NE)	3.5 (0.3–13.7)
OS						
Median, months (95% CI) ^¶^	14.5 (13.9–15.0)	13.4 (4.1–20.2)	10.3 (4.4–19.8)	12.7 (9.8–14.4)	14.2 (8.0–NE)	12.8 (10.2–14.2)
Event-free rate, % (95% CI) ^#^						
6 months	100.0 (NE–NE)	85.7 (33.4–97.9)	66.7 (28.2–87.8)	89.3 (74.0–95.9)	100.0 (NE–NE)	87.3 (76.3–93.5)
12 months	100.0 (NE–NE)	57.1 (17.2–83.7)	33.3 (7.8–62.3)	54.1 (37.0–68.5)	71.4 (25.8–92.0)	55.0 (41.9–66.4)

Data cutoff: 13 April 2021. * The efficacy analysis set was defined as all patients in the safety analysis set ^§^ who had a tumor assessment at baseline and at EOT unless they had discontinued treatment or the study early due to PD or death prior to tumor assessment. ^†^ Durable SD was defined as lasting ≥24 weeks. ^‡^ Medians were estimated by the Kaplan–Meier method, with 95% CIs estimated using the method of Brookmeyer and Crowley. ^§^ The safety analysis set consisted of all patients who received any dose of any of the study treatments. ^¶^ Medians were estimated by Kaplan–Meier method; two-sided 95% CIs were estimated using the method of Brookmeyer and Crowley. ^#^ Event-free rates were estimated by the Kaplan–Meier method, with 95% CIs constructed using Greenwood’s formula. Abbreviations: CI, confidence interval; CR, complete response; DoR, duration of response; EOT, end of treatment; NA, not available; NE, not estimable; ORR, objective response rate; OS, overall survival; PD, progressive disease; PFS, progression-free survival; PR, partial response; RANO, Response Assessment in Neuro-Oncology; RT, radiotherapy; SD, stable disease; TMZ, temozolomide.

**Table 5:** Primary and secondary efficacy endpoints in recurrent/refractory patients.

Endpoint	Arm C: Dose- Escalation Phase		Arm C: Dose- Expansion Phase	Total Recurrent/ Refractory Glioblastoma
	Pamiparib + TMZ 20 mg Days 1–21	Pamiparib + TMZ 40 mg Days 1–21	Pamiparib + TMZ 60 mg Days 1–7	
Disease Response Analyses (Unconfirmed; Efficacy Analysis Set *)				
N	9	7	28	44
Best response per RANO criteria, n (%)				
CR	0	0	0	0
PR	0	2 (28.6)	4 (14.3)	6 (13.6)
SD	5 (55.6)	3 (42.9)	4 (14.3)	12 (27.3)
PD	4 (44.4)	2 (28.6)	18 (64.3)	24 (54.5)
NE	0	0	2 (7.1)	2 (4.5)
ORR, n (%) [95% CI]	0 [0.0–33.6]	2 (28.6) [3.7–71.0]	4 (14.3) [4.0–32.7]	6 (13.6) [5.2–27.4]
Disease control rate (CR + PR + SD), n (%) [95% CI]	5 (55.6) [21.2–86.3]	5 (71.4) [29.0–96.3]	8 (28.6) [13.2–48.7]	18 (40.9) [26.3–56.8]
Clinical benefit rate (CR + PR + durable ^†^ SD), n (%) [95% CI]	0 [0.0–33.6]	2 (28.6) [3.7–71.0]	5 (17.9) [6.1–36.9]	7 (15.9) [6.6–30.1]
DoR per RANO criteria				
Events, n (%)	0	2 (100.0)	2 (50.0)	4 (66.7)
Median, months (95% CI) ^‡^	NA	6.6 (2.0–11.2)	12.7 (5.6–NE)	11.1 (2.0–NE)
Survival analyses (safety analysis set ^§^)				
N	9	8	30	47
PFS				
Median, months (95% CI) ^¶^	1.8 (0.8–3.4)	2.7 (0.7–7.4)	1.9 (1.5–1.9)	1.9 (1.7–2.3)
Event-free rate, % (95% CI) ^#^				
6 months	0.0 (NE–NE)	28.6 (4.1–61.2)	19.6 (7.3–36.3)	17.2 (7.6–30.1)
12 months	0.0 (NE–NE)	14.3 (0.7–46.5)	19.6 (7.3–36.3)	14.8 (6.0–27.2)
OS				
Median, months (95% CI) ^¶^	6.0 (2.6–9.8)	8.6 (3.0–NE)	7.8 (6.2–10.7)	7.3 (6.2–9.8)
Event-free rate, % (95% CI) ^#^				
6 months	50.0 (15.2–77.5)	62.5 (22.9–86.1)	75.4 (55.2–87.5)	68.5 (52.6–80.0)
12 months	0.0 (NE–NE)	16.7 (0.9–50.8)	26.4 (11.7–43.7)	20.0 (9.5–33.3)

Data cutoff: 13 April 2021. * The efficacy analysis set was defined as all patients in the safety analysis set. ^§^ who had measurable disease at baseline and one or more postbaseline tumor assessment unless they had discontinued treatment or the study early due to PD or death prior to tumor assessment. ^†^ Durable SD was defined as lasting ≥24 weeks. ^‡^ Medians were estimated by the Kaplan–Meier method, with 95% CIs estimated using the method of Brookmeyer and Crowley. ^§^ The safety analysis set consisted of all patients who received any dose of any of the study treatments. ^¶^ Medians were estimated by the Kaplan–Meier method; two-sided 95% CIs were estimated using the method of Brookmeyer and Crowley. ^#^ Event-free rates were estimated by the Kaplan–Meier method, with 95% CIs constructed using Greenwood’s formula. Abbreviations: CI, confidence interval; CR, complete response; DoR, duration of response; NA, not available; NE, not estimable; ORR, objective response rate; OS, overall survival; PD, progressive disease; PFS, progression-free survival; PR, partial response; RANO, Response Assessment in Neuro-Oncology; SD, stable disease; TMZ, temozolomide.

## Data Availability

BeOne Medicines voluntarily shares anonymous data on completed studies responsibly and provides qualified scientific and medical researchers access to anonymous data and supporting clinical trial documentation for clinical trials in dossiers for medicines and indications after submission and approval in the United States, China, and Europe. Clinical trials supporting subsequent local approvals, new indications, or combination products are eligible for sharing once corresponding regulatory approvals are achieved. BeOne Medicines shares data only when permitted by applicable data privacy and security laws and regulations. In addition, data can only be shared when it is feasible to do so without compromising the privacy of study participants. Qualified researchers may submit data requests/research proposals for BeOne Medicines review and consideration through BeOne Medicines’ Clinical Trial Webpage at https://beonemedicines.com/science/clinical-trials/.

## Supplementary Materials

The following supporting information can be downloaded at https://www.mdpi.com/article/10.3390/curroncol32100541/s1, Supplementary Methods: Inclusion criteria and exclusion criteria; Table S1: List of institutional review board or ethics committees; Table S2: Newly diagnosed glioblastoma (Arm A): overview of TEAEs (safety analysis set); Table S3: Newly diagnosed glioblastoma (Arm B): overview of TEAEs (safety analysis set); Table S4: Recurrent/refractory glioblastoma (Arm C): overview of TEAEs (safety analysis set); Table S5: Newly diagnosed glioblastoma (Arm A): TEAEs by system organ class and preferred term in ≥5% of patients (in the “all patients” column) (safety analysis set); Table S6: Newly diagnosed glioblastoma (Arm B): TEAEs by system organ class and preferred term in two or more patients (safety analysis set); Table S7: Recurrent/refractory glioblastoma (Arm C): TEAEs by system organ class and preferred term in ≥5% of patients (in “all patients” column) (safety analysis set); Table S8: Newly diagnosed glioblastoma (Arm A): grade ≥3 TEAEs by system organ class and preferred term (safety analysis set); Table S9: Newly diagnosed glioblastoma (Arm B): grade ≥3 TEAEs by system organ class and preferred term (safety analysis set); Table S10: Recurrent/refractory glioblastoma (Arm C): grade ≥ 3 TEAEs by system organ class and preferred term (safety analysis set).

## Abbreviations

The following abbreviations are used in this manuscript:

AE	Adverse event
BID	Twice daily
CI	Confidence interval
CR	Complete response
CTCAE	Common Terminology Criteria for Adverse Events
DCR	Disease control rate
DLT	Dose-limiting toxicity
DoR	Duration of response
ECOG PS	Eastern Cooperative Oncology Group performance status
EOT	End-of-treatment
*IDH*	Isocitrate dehydrogenase
*MGMT*	O-6-methylguanine-DNA methyltransferase
MRI	Magnetic resonance imaging
ORR	Objective response rate
OS	Overall survival
PARP	Poly (ADP-ribose) polymerase
PD	Progressive disease
PFS	Progression-free survival
PR	Partial response
QD	Once daily
RANO	Response Assessment in Neuro-Oncology
RT	Radiotherapy
SAE	Serious adverse event
SD	Stable disease
TEAE	Treatment-emergent adverse event
TMZ	Temozolomide
WHO	World Health Organization
