# Supplementary material for: Phase Ib/II Study of Pamiparib Plus Radiation Therapy and/or Temozolomide in Adult Patients with Treatment-Naïve or Recurrent/Refractory Glioblastoma
Source: Curr Oncol. 2025 Sep 27;32(10):541. doi: 10.3390/curroncol32100541 (PMC12562933; doi:10.3390/curroncol32100541)
Supplement: Supplementary file 1 [file curroncol-32-00541-s001.zip › curroncol-3810279-supplementary.pdf]

Phase Ib/II Study of Pamiparib Plus Radiation Therapy and/or Temozolomide in Adult Patients with Treatment-naïve or Recurrent/Refractory Glioblastoma

Supplementary Material

1. Supplementary Methods

1.1. Inclusion Criteria

1.1.1. For All Patients

1. Signed informed consent form
2. Age ≥18 years
3. Histopathologically confirmed glioblastoma (World Health Organization [WHO] Grade IV)
    - Tumor must have a supratentorial component
4. Agreement to provide archival tumor tissue for exploratory biomarker analyses
    - Collection of tumor tissue is strongly encouraged during the dose-escalation phase and will be mandatory during the dose-expansion phase for central confirmation of *MGMT* status and to support exploratory biomarker analyses
5. Ability to undergo serial magnetic resonance imaging (MRI) scans (computed tomography [CT] cannot substitute for MRI)
6. Brain MRI scan ≤14 days prior to day 1
    - Patients requiring glucocorticoids must be on a daily dose equivalent of dexamethasone 4 mg twice daily or less that has been stable for ≥7 days prior to the MRI
7. Eastern Cooperative Oncology Group (ECOG) performance status ≤1
8. Ability to swallow whole capsules
9. Adequate hematologic and end-organ function, as defined by the following laboratory results (obtained ≤2 weeks prior to day 1):
    - Absolute neutrophil count ≥1.5 × 10^9/L
    - Platelet count ≥100 × 10^9/L
    - Hemoglobin ≥9 g/dL ≥14 days after growth factor support or transfusion if appropriate
    - Serum creatinine ≤1.5 × upper limit of normal (ULN) or estimated creatinine clearance ≥50 mL/min (calculated using the institutional standard method)
    - Total serum bilirubin ≤1.5 × ULN (≤4 × ULN, if Gilbert's syndrome)
    - Aspartate and alanine aminotransferase (AST and ALT) ≤3 × ULN
    - Albumin ≥3 g/dL
    - International Normalized Ratio (INR) ≤1.5 and activated partial thromboplastin time (aPTT) ≤1.5 × ULN
10. Female patients of childbearing potential and female partners of male study patients must agree to practice highly effective methods of birth control for the duration of the study, and for ≥6 months after last study treatment. In addition, non-sterile male patients must agree to practice highly effective methods of birth control and avoid sperm donation for the duration of the study and for ≥6 months after the last dose of study drug
11. Willingness and ability to comply with all protocol-specified requirements

1.1.2. For Patients in Arms A and B (NOT Applicable to Arm C)

12. No previous treatment except surgery (i.e., no previous radiotherapy [RT], local chemotherapy, or systemic therapy for lower grade central nervous system tumors)
13. Ability to initiate RT ≤49 days after surgery, but ≥14 days after a biopsy or ≥28 days after an open biopsy or a craniotomy with adequate wound healing
14. Documentation of unmethylated *MGMT* promoter status
    - In escalation cohorts, it is preferable to determine *MGMT* status by quantitative methylation-specific polymerase chain reaction (MS-PCR). Other acceptable platforms include pyrosequencing methodologies and methylation sensitive high-resolution melting (MS-HRM) assays with comparable sensitivity, applied to archival or fresh tumor tissue. Sponsor must be notified prior to utilizing alternate assays or if data from acceptable alternate platforms are available
    - In expansion cohorts, archival or fresh tumor tissue must be submitted for central analysis of *MGMT* status

1.1.3. For Patients in Arm C Escalation (NOT Applicable to Arms A and B)

15. Documentation of *MGMT* promoter status
    - It is preferable to determine *MGMT* status by MS-PCR. Other acceptable platforms include pyrosequencing methodologies and MS-HRM assays with comparable sensitivity, applied to archival or fresh tumor tissue. Sponsor must be notified prior to utilizing alternate assays or if data from acceptable alternate platforms are available
16. No prior systemic chemotherapy, other than TMZ, for glioblastoma (including investigational cytotoxic chemotherapy) and no prior anti-angiogenic therapy
    - Prior use of Optune device is allowed with a minimum of 1 week since last Tumor Treating Fields application
17. Histologically confirmed secondary glioblastoma will be allowed during the dose escalation phase only
18. Disease that is evaluable or measurable as defined by Response Assessment in Neuro-Oncology (RANO) criteria

1.1.4. For Patients in Arm C Expansion

19. Histologically confirmed de novo (primary) glioblastoma with unequivocal first progressive disease (PD) after RT with concurrent/adjuvant TMZ chemotherapy as defined by one or more of the following:
    - PD ≥3 months after the end of RT
    - PD that is clearly outside the radiation field
    - PD that has been unequivocally proven by surgery/biopsy
20. Disease that is measurable as defined by RANO criteria
    - Patients with recurrent disease must have at least one bi-dimensionally measurable contrast-enhancing lesion with clearly defined margins by MRI scan, with minimal diameters of 10 mm, visible on two or more axial slices
21. Documentation of *MGMT* promoter status
    - Tumor tissue (archival or fresh) must be submitted for central analysis of *MGMT* status
    - Patients will be enrolled into one of two expansion cohorts based on *MGMT* methylation status

1.2. Exclusion Criteria

1.2.1. For All Patients

1. Chemotherapy, biologic therapy, immunotherapy, or investigational agent ≤21 days (or ≤5 half-lives, whichever is shorter) prior to day 1
2. Unresolved acute effects of any prior therapy of Grade ≥2, except for adverse events (AEs) not constituting safety risk by investigator judgement
3. Major surgical procedure, open biopsy, or significant traumatic injury ≤28 days prior to day 1, or anticipation of need for major surgical procedure during the course of the study
    - Placement of vascular access device is not considered major surgery
4. Other diagnosis of malignancy
    - Except for surgically excised non-melanoma skin cancer, adequately treated carcinoma in situ of the cervix, localized prostate cancer treated with curative intent, adequately treated low-stage bladder cancer, ductal carcinoma in situ treated surgically with curative intent, or a malignancy diagnosed >2 years ago with no current evidence of disease and no therapy ≤2 years prior to day 1
5. Active infection requiring systemic treatment
6. Have known HIV infection or serologic status reflecting active viral hepatitis infection:
    - Patients with untreated chronic hepatitis B or chronic hepatitis B virus (HBV) carriers whose HBV DNA is >500 IU/mL or patients with active hepatitis C virus should be excluded. Note: Inactive hepatitis B surface antigen carriers, treated and stable hepatitis B (HBV DNA ≤500 IU/mL), and cured patients with hepatitis C can be enrolled
7. Any of the following cardiovascular criteria:
    - Current evidence of cardiac ischemia
    - Current symptomatic pulmonary embolism
    - Acute myocardial infarction ≤6 months prior to day 1
    - Heart failure of New York Heart Association Classification III or IV ≤6 months prior to day 1
    - Grade ≥2 ventricular arrhythmia ≤6 months prior to day 1
    - Cerebral vascular accident or transient ischemic attack ≤6 months prior to day 1
8. Active inflammatory gastrointestinal disease, chronic diarrhea, known diverticular disease, or previous gastric resection or lap-band surgery
    - Gastroesophageal reflux disease under treatment with proton-pump inhibitors is allowed (assuming no drug interaction potential)
9. Active bleeding disorder, including gastrointestinal bleeding, as evidenced by hematemesis, significant hemoptysis, or melena ≤6 months prior to day 1
10. Anticoagulation with heparin, warfarin, or other anticoagulants other than the following:
    - Low-dose aspirin and/or non-steroidal anti-inflammatory agents are allowed
    - Use of thrombolytics to establish patency of indwelling venous catheters is allowed
    - Prophylactic anticoagulation for venous access devices is allowed as long as INR is ≤1.5 and aPTT is ≤1.5 institutional ULN
    - Low molecular weight heparin for treatment of thromboembolic events
11. Use ≤10 days (or ≤5 half-lives, whichever is shorter) prior to day 1 or anticipated need for food or drugs known to be strong or moderate CYP3A inhibitors or strong CYP3A inducers including known enzyme inducing antiepileptic drugs
12. Pregnancy or nursing
    - Females of childbearing potential require a negative serum pregnancy test ≤7 days before day 1
13. Significant intercurrent illness that may result in the patient's death prior to death from glioblastoma

14. Known history of intolerance to the excipients of the pamiparib capsule

1.2.2. For Patients in Arms B and C (NOT Applicable to Arm A)

15. Known hypersensitivity to any temozolomide component or to dacarbazine
16. Have hereditary problems of galactose intolerance, the Lapp lactase deficiency, or glucose-galactose malabsorption

Supplementary Table S1. List of institutional review board or ethics committees.

Country	Site No.	Site Name	Principal Investigator	Authority/Committee Name
United States	0101	Center for Neurosciences	Badruddoja, Michael	WIRB
United States	0102	Dana-Farber Cancer Institute	Wen, Patrick	Dana Farber Cancer Institute
United States	0103	Henry Ford Hospital	Walbert, Tobias	Henry Ford Health Systems IRB
United States	0114	Sarah Cannon Research Institute at Health One	Pearlman, Michael	IntegReview IRB
United States	0115	Memorial Sloan Kettering Cancer Center	Piotrowski, Anna	Memorial Sloan-Kettering Cancer Center IRB
United States	0119	Thomas Jefferson University	Glass, Jon	WIRB
United States	0120	UCLA	Cloughesy, Timothy	UCLA Medical Center IRB
United States	0121	Massachusetts General Hospital	Wen, Patrick	Office for Human Research Studies
United States	0128	Tennessee Oncology, PLLC - Nashville	Shih, Kent	IntegReview IRB
United States	0129	HCA Midwest Health Kansas City - Sarah Cannon	Kalra, Amandeep	IntegReview IRB
United States	0130	Stephenson Cancer Center	Battiste, James	IntegReview IRB
United States	0132	University of California San Francisco (PARENT)	Butowski, Nicholas	University of California IRB
United States	0133	University of Colorado	Ney, Doug	WIRB
United States	0137	The Rector and Visitors of the University of Virginia	Schiff, David	UVA IRB-HSR
United States	0140	Washington University	Campian, Jian Li	Washington University in St. Louis IRB

United States	0149	Huntsman Cancer Institute	Colman, Howard	Huntsville Hospital Institutional Review Committee
United States	0150	Cleveland Clinic Foundation	Ahluwalia, Manmeet	Cleveland Clinic IRB
United States	0151	Yale Cancer Center	Becker, Kevin	Yale University IRB
United States	0152	University of Washington	Taylor, Lynne	WIRB
United States	0153	The Ohio State University	Giglio, Pierre Vinay	WIRB
United States	0156	Penn State University	Aregawi, Dawit	Penn State Milton S. Hershey Medical Center IRB
United States	0178	Columbia University Hervert Irving Comprehensive Cancer Center	Lassman, Andrew	Columbia University IRB
Netherlands	3103	Universitair Medisch Centrum Groningen	van den Bent, Martin	Stichting Beoordeling Ethiek Biomedisch Onderzoek
Netherlands	3104	Universitair Medisch Centrum Groningen	Walenkamp, Annemiek	Stichting Beoordeling Ethiek Biomedisch Onderzoek
France	3304	Institut Gustave Roussy	Massard, Christophe	Comité de Protection des Personnes Sud-Méditerranée IV
France	3306	Hopital Claude Huriez - CHU Lille	Le Rhun, Emilie	Comité de Protection des Personnes Sud-Méditerranée IV
France	3307	CHU Bordeaux	Ravaud, Alain	Comité de Protection des Personnes Sud-Méditerranée IV
Switzerland	4101	Universitätsspital Zuerich	Weller, Michael	Kantonale Ethikkommission Zürich (KEK-Zürich)
Switzerland	4102	Universitaetsspital Basel	Laeubli, Heinz	Ethikkommission der Nordwest- und Zentralschweiz (EKNZ)
Switzerland	4103	Istituto Oncologico della Svizzera Italiana (IOSI)- Ente Ospedaliero Cantonale (EOC)	Pesce, Gianfranco	Kantonale Ethikkommission Zürich (KEK-Zürich)

United States	4113	MD Anderson Cancer Center	Kamiya Matsuoka, Carlos	MD Anderson Cancer Center IRB
United Kingdom	4408	University College London Hospitals	Mulholland, Paul	
United Kingdom	4421	Royal Marsden Hospital-Sutton	Lopez, Juanita	
United Kingdom	4422	Beatson West of Scotland Cancer Centre	Chalmers, Anthony	
Australia	6101	Austin Health	Gan, Hui	Austin Health Human Research Ethics Committee
Australia	6118	Prince of Wales Hospital	Hovey, Elizabeth	South Eastern Sydney LHD Human Research Ethics Committee (RGO)
Australia	6123	Royal North Shore Hospital	Khasraw, Mustafa	Austin Health Human Research Ethics Committee
Australia	6133	Liverpool Hospital	De Souza, Paul	Austin Health Human Research Ethics Committee

Supplementary Table S2. Newly diagnosed glioblastoma (Arm A): overview of TEAEs (safety analysis set).

	Arm A: Dose-escalation Phase			Arm A: Dose-expansion Phase	
Events, n (%)	**Pamiparib 2 Weeks + RT (N = 3)**	**Pamiparib 4 Weeks + RT (N = 8)**	**Pamiparib 6 Weeks + RT (N = 9)**	**Pamiparib 6 Weeks + RT (N = 40)**	**All Patients (N = 60)**
Patients with ≥1 TEAE	3 (100.0)	8 (100.0)	9 (100.0)	40 (100.0)	60 (100.0)
TEAE with grade ≥3	1 (33.3)	3 (37.5)	4 (44.4)	25 (62.5)	33 (55.0)
Treatment-emergent SAEs	0 (0.0)	2 (25.0)	2 (22.2)	18 (45.0)	22 (36.7)
TEAE leading to death	0 (0.0)	0 (0.0)	0 (0.0)	3 (7.5)	3 (5.0)
TEAE leading to treatment discontinuation of pamiparib only	0 (0.0)	0 (0.0)	0 (0.0)	0 (0.0)	0 (0.0)
TEAE leading to treatment discontinuation of RT only	0 (0.0)	0 (0.0)	0 (0.0)	1 (2.5)	1 (1.7)
TEAE leading to treatment discontinuation of TMZ (maintenance) only	0 (0.0)	0 (0.0)	0 (0.0)	0 (0.0)	0 (0.0)
TEAE leading to treatment discontinuation of both pamiparib and RT	0 (0.0)	1 (12.5)	2 (22.2)	1 (2.5)	4 (6.7)
TEAE leading to treatment discontinuation of both pamiparib and TMZ (maintenance)	0 (0.0)	0 (0.0)	0 (0.0)	1 (2.5)	1 (1.7)
TEAE leading to dose modification of pamiparib only	0 (0.0)	0 (0.0)	0 (0.0)	12 (30.0)	12 (20.0)
Leading to dose interruption	0 (0.0)	0 (0.0)	0 (0.0)	12 (30.0)	12 (20.0)
Leading to dose reduction	0 (0.0)	0 (0.0)	0 (0.0)	0 (0.0)	0 (0.0)
TEAE leading to dose modification of RT only	0 (0.0)	1 (12.5)	0 (0.0)	3 (7.5)	4 (6.7)
Leading to dose interruption	0 (0.0)	1 (12.5)	0 (0.0)	3 (7.5)	4 (6.7)
Leading to dose reduction	0 (0.0)	0 (0.0)	0 (0.0)	0 (0.0)	0 (0.0)
TEAE leading to dose modification of TMZ (maintenance) only	0 (0.0)	0 (0.0)	0 (0.0)	3 (7.5)	3 (5.0)
Leading to dose interruption	0 (0.0)	0 (0.0)	0 (0.0)	2 (5.0)	2 (3.3)
Leading to dose reduction	0 (0.0)	0 (0.0)	0 (0.0)	1 (2.5)	1 (1.7)

Events, n (%)	Arm A: Dose-escalation Phase Pamiparib 2 Weeks + RT (N = 3)	Arm A: Dose-escalation Phase Pamiparib 4 Weeks + RT (N = 8)	Arm A: Dose-escalation Phase Pamiparib 6 Weeks + RT (N = 9)	Arm A: Dose-expansion Phase Pamiparib 6 Weeks + RT (N = 40)	All Patients (N = 60)
TEAE leading to dose modification of both pamiparib and RT	0 (0.0)	1 (12.5)	0 (0.0)	3 (7.5)	4 (6.7)
Leading to dose interruption	0 (0.0)	1 (12.5)	0 (0.0)	3 (7.5)	4 (6.7)
Leading to dose reduction	0 (0.0)	0 (0.0)	0 (0.0)	0 (0.0)	0 (0.0)
TEAE leading to dose modification of both pamiparib and TMZ (maintenance)	0 (0.0)	0 (0.0)	0 (0.0)	15 (37.5)	15 (25.0)
Leading to dose interruption	0 (0.0)	0 (0.0)	0 (0.0)	15 (37.5)	15 (25.0)
Leading to dose reduction	0 (0.0)	0 (0.0)	0 (0.0)	0 (0.0)	0 (0.0)
TEAE related to pamiparib only	1 (33.3)	5 (62.5)	7 (77.8)	25 (62.5)	38 (63.3)
TEAE related to RT only	1 (33.3)	5 (62.5)	5 (55.6)	28 (70.0)	39 (65.0)
TEAE related to TMZ (maintenance) only	0 (0.0)	0 (0.0)	0 (0.0)	5 (12.5)	5 (8.3)
TEAE related to both pamiparib and RT	3 (100.0)	2 (25.0)	4 (44.4)	29 (72.5)	38 (63.3)
TEAE related to both pamiparib and TMZ (maintenance)	0 (0.0)	1 (12.5)	0 (0.0)	19 (47.5)	20 (33.3)
TEAE related to pamiparib only grade ≥3	0 (0.0)	1 (12.5)	2 (22.2)	2 (5.0)	5 (8.3)
TEAE related to RT only grade ≥3	0 (0.0)	0 (0.0)	0 (0.0)	2 (5.0)	2 (3.3)
TEAE related to TMZ (maintenance) only grade ≥3	0 (0.0)	0 (0.0)	0 (0.0)	0 (0.0)	0 (0.0)
TEAE related to both pamiparib and RT grade ≥3	0 (0.0)	0 (0.0)	1 (11.1)	3 (7.5)	4 (6.7)
TEAE related to both pamiparib and TMZ (maintenance) grade ≥3	0 (0.0)	0 (0.0)	0 (0.0)	9 (22.5)	9 (15.0)
Serious TEAE related to pamiparib only	0 (0.0)	0 (0.0)	1 (11.1)	2 (5.0)	3 (5.0)
Serious TEAE related to RT only	0 (0.0)	0 (0.0)	0 (0.0)	1 (2.5)	1 (1.7)
Serious TEAE related to TMZ (maintenance) only	0 (0.0)	0 (0.0)	0 (0.0)	0 (0.0)	0 (0.0)
Serious TEAE related to both pamiparib and RT	0 (0.0)	0 (0.0)	0 (0.0)	1 (2.5)	1 (1.7)
Serious TEAE related to both pamiparib and TMZ (maintenance)	0 (0.0)	0 (0.0)	0 (0.0)	2 (5.0)	2 (3.3)
Treatment-related TEAEs leading to death	0 (0.0)	0 (0.0)	0 (0.0)	0 (0.0)	0 (0.0)

Events, n (%)	Arm A: Dose-escalation Phase Pamiparib 2 Weeks + RT (N = 3)	Pamiparib 4 Weeks + RT (N = 8)	Pamiparib 6 Weeks + RT (N = 9)	Arm A: Dose-expansion Phase Pamiparib 6 Weeks + RT (N = 40)	All Patients (N = 60)
Treatment-related TEAE leading to treatment discontinuation of pamiparib only	0 (0.0)	0 (0.0)	0 (0.0)	0 (0.0)	0 (0.0)
Treatment-related TEAE leading to treatment discontinuation of RT only	0 (0.0)	0 (0.0)	0 (0.0)	0 (0.0)	0 (0.0)
Treatment-related TEAE leading to treatment discontinuation of TMZ (maintenance) only	0 (0.0)	0 (0.0)	0 (0.0)	0 (0.0)	0 (0.0)
Treatment-related TEAE leading to treatment discontinuation of both pamiparib and RT	0 (0.0)	0 (0.0)	2 (22.2)	0 (0.0)	2 (3.3)
Treatment-related TEAE leading to treatment discontinuation of both pamiparib and TMZ (maintenance)	0 (0.0)	0 (0.0)	0 (0.0)	0 (0.0)	0 (0.0)
Treatment-related TEAE leading to dose modification of pamiparib only	0 (0.0)	0 (0.0)	0 (0.0)	7 (17.5)	7 (11.7)
Leading to dose interruption	0 (0.0)	0 (0.0)	0 (0.0)	7 (17.5)	7 (11.7)
Leading to dose reduction	0 (0.0)	0 (0.0)	0 (0.0)	0 (0.0)	0 (0.0)
Treatment-related TEAE leading to dose modification of RT only	0 (0.0)	1 (12.5)	0 (0.0)	0 (0.0)	1 (1.7)
Leading to dose interruption	0 (0.0)	1 (12.5)	0 (0.0)	0 (0.0)	1 (1.7)
Leading to dose reduction	0 (0.0)	0 (0.0)	0 (0.0)	0 (0.0)	0 (0.0)
Treatment-related TEAE leading to dose modification of TMZ (maintenance) only	0 (0.0)	0 (0.0)	0 (0.0)	3 (7.5)	3 (5.0)
Leading to dose interruption	0 (0.0)	0 (0.0)	0 (0.0)	2 (5.0)	2 (3.3)
Leading to dose reduction	0 (0.0)	0 (0.0)	0 (0.0)	1 (2.5)	1 (1.7)
Treatment-related TEAE leading to dose modification of both pamiparib and RT	0 (0.0)	0 (0.0)	0 (0.0)	1 (2.5)	1 (1.7)
Leading to dose interruption	0 (0.0)	0 (0.0)	0 (0.0)	1 (2.5)	1 (1.7)
Leading to dose reduction	0 (0.0)	0 (0.0)	0 (0.0)	0 (0.0)	0 (0.0)

Events, n (%)	Arm A: Dose-escalation Phase Pamiparib 2 Weeks + RT (N = 3)	Arm A: Dose-escalation Phase Pamiparib 4 Weeks + RT (N = 8)	Arm A: Dose-escalation Phase Pamiparib 6 Weeks + RT (N = 9)	Arm A: Dose-expansion Phase Pamiparib 6 Weeks + RT (N = 40)	All Patients (N = 60)
Treatment-related TEAE leading to dose modification of both pamiparib and TMZ (maintenance)	0 (0.0)	0 (0.0)	0 (0.0)	12 (30.0)	12 (20.0)
Leading to dose interruption	0 (0.0)	0 (0.0)	0 (0.0)	12 (30.0)	12 (20.0)
Leading to dose reduction	0 (0.0)	0 (0.0)	0 (0.0)	0 (0.0)	0 (0.0)

Data cutoff: 13 April 2021.

Note: For each row category, a patient with two or more AEs in that category is counted only once. Treatment-related TEAEs are TEAEs that are considered by the investigator to be possibly or probably related to study drug or with missing assessment of the causal relationship. A treatment-related AE refers to an AE related to any of study treatment of pamiparib, RT, or TMZ. AE grades are evaluated based on CTCAE (version 4.03).

Abbreviations: AE, adverse event; CTCAE, Common Terminology Criteria for Adverse Events; RT, radiation therapy; SAE, serious adverse event; TEAE, treatment-emergent adverse event; TMZ, temozolomide.

Supplementary Table S3. Newly-diagnosed glioblastoma (Arm B): overview of TEAEs (safety analysis set).

Events, N (%)	Dose-escalation Phase<br>Pamiparib 6 Weeks + RT + TMZ 60 mg Weeks 1 and 5 (N = 9)
Patients with ≥1 TEAE	9 (100.0)
TEAE with grade ≥3	4 (44.4)
Treatment-emergent SAEs	2 (22.2)
TEAE leading to death	0 (0.0)
TEAE leading to treatment discontinuation of pamiparib only	0 (0.0)
TEAE leading to treatment discontinuation of RT only	0 (0.0)
TEAE leading to treatment discontinuation of TMZ only	0 (0.0)
TEAE leading to treatment discontinuation of both pamiparib and RT	0 (0.0)
TEAE leading to treatment discontinuation of both pamiparib and TMZ	1 (11.1)
TEAE leading to treatment discontinuation of pamiparib, RT and TMZ	0 (0.0)
TEAE leading to dose modification of pamiparib only	2 (22.2)
Leading to dose interruption	2 (22.2)
Leading to dose reduction	0 (0.0)
TEAE leading to dose modification of RT only	0 (0.0)
Leading to dose interruption	0 (0.0)
Leading to dose reduction	0 (0.0)
TEAE leading to dose modification of TMZ only	1 (11.1)
Leading to dose interruption	1 (11.1)
Leading to dose reduction	0 (0.0)
TEAE leading to dose modification of both pamiparib and TMZ	4 (44.4)
Leading to dose interruption	3 (33.3)
Leading to dose reduction	0 (0.0)
TEAE leading to dose modification of pamiparib, RT, and TMZ	0 (0.0)
Leading to dose interruption	0 (0.0)
Leading to dose reduction	0 (0.0)
TEAE related to pamiparib only	3 (33.3)
TEAE related to RT only	6 (66.7)

Events, N (%)	Dose-escalation Phase<br>Pamiparib 6 Weeks + RT + TMZ 60 mg Weeks 1 and 5 (N = 9)
TEAE related to TMZ only	5 (55.6)
TEAE related to both pamiparib and TMZ	6 (66.7)
TEAE related to pamiparib, RT, and TMZ	4 (44.4)
TEAE related to pamiparib only grade ≥3	1 (11.1)
TEAE related to RT only grade ≥3	1 (11.1)
TEAE related to TMZ only grade ≥3	0 (0.0)
TEAE related to both pamiparib and TMZ grade ≥3	3 (33.3)
TEAE related to pamiparib, RT and TMZ grade ≥3	1 (11.1)
Serious TEAE related to pamiparib only	0 (0.0)
Serious TEAE related to RT only	0 (0.0)
Serious TEAE related to TMZ only	0 (0.0)
Serious TEAE related to both pamiparib and TMZ	1 (11.1)
Serious TEAE related to pamiparib, RT, and TMZ	1 (11.1)
Treatment-related TEAEs leading to death	0 (0.0)
Treatment-related TEAE leading to treatment discontinuation of pamiparib only	0 (0.0)
Treatment-related TEAE leading to treatment discontinuation of RT only	0 (0.0)
Treatment-related TEAE leading to treatment discontinuation of TMZ only	0 (0.0)
Treatment-related TEAE leading to treatment discontinuation of both pamiparib and TMZ	1 (11.1)
Treatment-related TEAE leading to treatment discontinuation of pamiparib, RT and TMZ	0 (0.0)
Treatment-related TEAE leading to dose modification of pamiparib only	2 (22.2)
Leading to dose interruption	2 (22.2)
Leading to dose reduction	0 (0.0)
Treatment-related TEAE leading to dose modification of RT only	0 (0.0)
Leading to dose interruption	0 (0.0)
Leading to dose reduction	0 (0.0)
Treatment-related TEAE leading to dose modification of TMZ only	1 (11.1)
Leading to dose interruption	1 (11.1)
Leading to dose reduction	0 (0.0)
Treatment-related TEAE leading to dose modification of both pamiparib and TMZ	4 (44.4)

Events, N (%)	Dose-escalation Phase<br>Pamiparib 6 Weeks + RT + TMZ 60 mg Weeks 1 and 5 (N = 9)
Leading to dose interruption	3 (33.3)
Leading to dose reduction	0 (0.0)
Treatment-related TEAE leading to dose modification of pamiparib, RT and TMZ	0 (0.0)
Leading to dose interruption	0 (0.0)
Leading to dose reduction	0 (0.0)

Data cutoff: 13 April 2021.

Note: For each row category, a patient with two or more AEs in that category is counted only once. Treatment-related TEAEs are TEAEs that are considered by the investigator to be possibly or probably related to study drug or with missing assessment of the causal relationship. A treatment-related AE refers to an AE related to any of study treatment of pamiparib, RT, or TMZ. Adverse event grades are evaluated based on CTCAE (version 4.03).

Abbreviations: AE, adverse event; CTCAE, Common Terminology Criteria for Adverse Events; RT, radiation therapy; SAE, serious adverse event; TEAE, treatment-emergent adverse event; TMZ, temozolomide.

Supplementary Table S4. Recurrent/refractory glioblastoma (Arm C): overview of TEAEs (safety analysis set).

	Dose-escalation Phase		Dose-expansion Phase	
Events, N (%)	Pamiparib + TMZ 20 mg Days 1–21 (*N* = 9)	Pamiparib + TMZ 40 mg Days 1–21 (N = 8)	Pamiparib + TMZ 60 mg Dasy 1–7 (N = 30)	All Patients (N = 47)
Patients with ≥1 TEAE	9 (100.0)	8 (100.0)	29 (96.7)	46 (97.9)
TEAE with grade ≥3	5 (55.6)	7 (87.5)	19 (63.3)	31 (66.0)
Treatment-emergent SAEs	4 (44.4)	3 (37.5)	11 (36.7)	18 (38.3)
TEAE leading to death	0 (0.0)	0 (0.0)	1 (3.3)	1 (2.1)
TEAE leading to treatment discontinuation of pamiparib only	0 (0.0)	0 (0.0)	0 (0.0)	0 (0.0)
TEAE leading to treatment discontinuation of TMZ only	0 (0.0)	0 (0.0)	0 (0.0)	0 (0.0)
TEAE leading to treatment discontinuation of both pamiparib and TMZ	1 (11.1)	2 (25.0)	3 (10.0)	6 (12.8)
TEAE leading to dose modification of pamiparib only	1 (11.1)	2 (25.0)	10 (33.3)	13 (27.7)
Leading to dose interruption	1 (11.1)	2 (25.0)	10 (33.3)	13 (27.7)
Leading to dose reduction	0 (0.0)	0 (0.0)	0 (0.0)	0 (0.0)
TEAE leading to dose modification of TMZ only	0 (0.0)	2 (25.0)	2 (6.7)	4 (8.5)
Leading to dose interruption	0 (0.0)	1 (12.5)	2 (6.7)	3 (6.4)
Leading to dose reduction	0 (0.0)	1 (12.5)	1 (3.3)	2 (4.3)
TEAE leading to dose modification of both pamiparib and TMZ	6 (66.7)	4 (50.0)	11 (36.7)	21 (44.7)
Leading to dose interruption	6 (66.7)	4 (50.0)	10 (33.3)	20 (42.6)
Leading to dose reduction	0 (0.0)	0 (0.0)	1 (3.3)	1 (2.1)
TEAE related to pamiparib only	2 (22.2)	3 (37.5)	6 (20.0)	11 (23.4)
TEAE related to TMZ only	3 (33.3)	1 (12.5)	12 (40.0)	16 (34.0)
TEAE related to both pamiparib and TMZ	6 (66.7)	6 (75.0)	22 (73.3)	34 (72.3)
TEAE related to pamiparib only grade ≥3	0 (0.0)	0 (0.0)	0 (0.0)	0 (0.0)
TEAE related to TMZ only grade ≥3	0 (0.0)	1 (12.5)	2 (6.7)	3 (6.4)
TEAE related to both pamiparib and TMZ grade ≥3	2 (22.2)	5 (62.5)	14 (46.7)	21 (44.7)
Serious TEAE related to pamiparib only	0 (0.0)	0 (0.0)	1 (3.3)	1 (2.1)

Events, N (%)	Dose-escalation Phase Pamiparib + TMZ 20 mg Days 1–21 (*N* = 9)	Dose-escalation Phase Pamiparib + TMZ 40 mg Days 1–21 (N = 8)	Dose-expansion Phase Pamiparib + TMZ 60 mg Dasy 1–7 (N = 30)	All Patients (N = 47)
Serious TEAE related to TMZ only	0 (0.0)	0 (0.0)	0 (0.0)	0 (0.0)
Serious TEAE related to both pamiparib and TMZ	1 (11.1)	2 (25.0)	1 (3.3)	4 (8.5)
Treatment-related TEAEs leading to death	0 (0.0)	0 (0.0)	0 (0.0)	0 (0.0)
Treatment-related TEAE leading to treatment discontinuation of pamiparib only	0 (0.0)	0 (0.0)	0 (0.0)	0 (0.0)
Treatment-related TEAE leading to treatment discontinuation of TMZ only	0 (0.0)	0 (0.0)	0 (0.0)	0 (0.0)
Treatment-related TEAE leading to treatment discontinuation of both pamiparib and TMZ	1 (11.1)	2 (25.0)	0 (0.0)	3 (6.4)
Treatment-related TEAE leading to dose modification of pamiparib only	0 (0.0)	2 (25.0)	8 (26.7)	10 (21.3)
Leading to dose interruption	0 (0.0)	2 (25.0)	8 (26.7)	10 (21.3)
Leading to dose reduction	0 (0.0)	0 (0.0)	0 (0.0)	0 (0.0)
Treatment-related TEAE leading to dose modification of TMZ only	0 (0.0)	2 (25.0)	2 (6.7)	4 (8.5)
Leading to dose interruption	0 (0.0)	1 (12.5)	2 (6.7)	3 (6.4)
Leading to dose reduction	0 (0.0)	1 (12.5)	1 (3.3)	2 (4.3)
Treatment-related TEAE leading to dose modification of both pamiparib and TMZ	4 (44.4)	4 (50.0)	9 (30.0)	17 (36.2)
Leading to dose interruption	4 (44.4)	4 (50.0)	8 (26.7)	16 (34.0)
Leading to dose reduction	0 (0.0)	0 (0.0)	1 (3.3)	1 (2.1)

Data cutoff: 13 April 2021.

Note: For each row category, a patient with two or more AEs in that category is counted only once. Treatment-related TEAEs are TEAEs that are considered by the investigator to be possibly or probably related to study drug or with missing assessment of the causal relationship. A treatment-related AE refers to an AE related to any of study treatment of pamiparib or TMZ.

Abbreviations: AE, adverse event; SAE, serious adverse event; TEAE, treatment-emergent adverse event; TMZ, temozolomide.

Supplementary Table S5. Newly-diagnosed glioblastoma (Arm A): TEAEs by system organ class and preferred term in ≥5% of patients (in the "all patients" column) (safety analysis set).

	Arm A: Dose-escalation Phase			Arm A: Dose-expansion Phase	
**System Organ Class Preferred Term, N (%)**	**Pamiparib 2 Weeks + RT (N = 3)**	**Pamiparib 4 Weeks + RT (N = 8)**	**Pamiparib 6 Weeks + RT (N = 9)**	**Pamiparib 6 Weeks + RT (N = 40)**	**All Patients (N = 60)**
Patients with ≥1 TEAE	3 (100.0)	8 (100.0)	9 (100.0)	40 (100.0)	60 (100.0)
Gastrointestinal disorders	2 (66.7)	7 (87.5)	8 (88.9)	37 (92.5)	54 (90.0)
Nausea	1 (33.3)	3 (37.5)	5 (55.6)	29 (72.5)	38 (63.3)
Constipation	0 (0.0)	1 (12.5)	3 (33.3)	14 (35.0)	18 (30.0)
Vomiting	1 (33.3)	0 (0.0)	3 (33.3)	13 (32.5)	17 (28.3)
Diarrhea	0 (0.0)	2 (25.0)	0 (0.0)	12 (30.0)	14 (23.3)
Abdominal pain	1 (33.3)	1 (12.5)	1 (11.1)	5 (12.5)	8 (13.3)
Dry mouth	0 (0.0)	0 (0.0)	0 (0.0)	4 (10.0)	4 (6.7)
Dyspepsia	0 (0.0)	1 (12.5)	1 (11.1)	2 (5.0)	4 (6.7)
Flatulence	0 (0.0)	0 (0.0)	1 (11.1)	2 (5.0)	3 (5.0)
Nervous system disorders	2 (66.7)	6 (75.0)	8 (88.9)	36 (90.0)	52 (86.7)
Headache	1 (33.3)	1 (12.5)	4 (44.4)	18 (45.0)	24 (40.0)
Dizziness	0 (0.0)	1 (12.5)	0 (0.0)	11 (27.5)	12 (20.0)
Aphasia	0 (0.0)	1 (12.5)	3 (33.3)	6 (15.0)	10 (16.7)
Dysgeusia	0 (0.0)	0 (0.0)	0 (0.0)	10 (25.0)	10 (16.7)
Hemiparesis	0 (0.0)	0 (0.0)	1 (11.1)	9 (22.5)	10 (16.7)
Seizure	0 (0.0)	0 (0.0)	1 (11.1)	8 (20.0)	9 (15.0)
Memory impairment	0 (0.0)	1 (12.5)	0 (0.0)	6 (15.0)	7 (11.7)
Partial seizures	0 (0.0)	1 (12.5)	1 (11.1)	3 (7.5)	5 (8.3)
Paresthesia	0 (0.0)	1 (12.5)	1 (11.1)	1 (2.5)	3 (5.0)
Visual field defect	0 (0.0)	0 (0.0)	0 (0.0)	3 (7.5)	3 (5.0)
General disorders and administration site conditions	3 (100.0)	4 (50.0)	7 (77.8)	31 (77.5)	45 (75.0)
Fatigue	3 (100.0)	2 (25.0)	6 (66.7)	29 (72.5)	40 (66.7)
Asthenia	0 (0.0)	1 (12.5)	1 (11.1)	2 (5.0)	4 (6.7)
Edema peripheral	0 (0.0)	0 (0.0)	0 (0.0)	4 (10.0)	4 (6.7)

System Organ Class Preferred Term, N (%)	Arm A: Dose-escalation Phase Pamiparib 2 Weeks + RT (N = 3)	Pamiparib 4 Weeks + RT (N = 8)	Pamiparib 6 Weeks + RT (N = 9)	Arm A: Dose-expansion Phase Pamiparib 6 Weeks + RT (N = 40)	All Patients (N = 60)
Gait disturbance	0 (0.0)	0 (0.0)	0 (0.0)	3 (7.5)	3 (5.0)
Malaise	0 (0.0)	0 (0.0)	0 (0.0)	3 (7.5)	3 (5.0)
Metabolism and nutrition disorders	2 (66.7)	2 (25.0)	5 (55.6)	20 (50.0)	29 (48.3)
Decreased appetite	1 (33.3)	1 (12.5)	3 (33.3)	14 (35.0)	19 (31.7)
Hyperglycemia	1 (33.3)	0 (0.0)	1 (11.1)	2 (5.0)	4 (6.7)
Hyponatremia	0 (0.0)	0 (0.0)	0 (0.0)	4 (10.0)	4 (6.7)
Hypokalemia	0 (0.0)	0 (0.0)	1 (11.1)	2 (5.0)	3 (5.0)
Hypophosphatemia	0 (0.0)	0 (0.0)	0 (0.0)	3 (7.5)	3 (5.0)
Skin and subcutaneous tissue disorders	2 (66.7)	3 (37.5)	2 (22.2)	20 (50.0)	27 (45.0)
Alopecia	2 (66.7)	2 (25.0)	2 (22.2)	14 (35.0)	20 (33.3)
Pruritus	1 (33.3)	1 (12.5)	0 (0.0)	1 (2.5)	3 (5.0)
Investigations	0 (0.0)	0 (0.0)	2 (22.2)	21 (52.5)	23 (38.3)
Weight decreased	0 (0.0)	0 (0.0)	1 (11.1)	9 (22.5)	10 (16.7)
Platelet count decreased	0 (0.0)	0 (0.0)	1 (11.1)	7 (17.5)	8 (13.3)
White blood cell count decreased	0 (0.0)	0 (0.0)	1 (11.1)	6 (15.0)	7 (11.7)
Neutrophil count decreased	0 (0.0)	0 (0.0)	0 (0.0)	6 (15.0)	6 (10.0)
Blood creatinine increased	0 (0.0)	0 (0.0)	0 (0.0)	4 (10.0)	4 (6.7)
Lymphocyte count decreased	0 (0.0)	0 (0.0)	1 (11.1)	3 (7.5)	4 (6.7)
Alanine aminotransferase increased	0 (0.0)	0 (0.0)	0 (0.0)	3 (7.5)	3 (5.0)
Aspartate aminotransferase increased	0 (0.0)	0 (0.0)	0 (0.0)	3 (7.5)	3 (5.0)
Psychiatric disorders	0 (0.0)	2 (25.0)	2 (22.2)	19 (47.5)	23 (38.3)
Anxiety	0 (0.0)	0 (0.0)	1 (11.1)	6 (15.0)	7 (11.7)
Confusional state	0 (0.0)	2 (25.0)	1 (11.1)	4 (10.0)	7 (11.7)
Insomnia	0 (0.0)	0 (0.0)	0 (0.0)	6 (15.0)	6 (10.0)

System Organ Class<br>Preferred Term, N (%)	Arm A: Dose-escalation Phase<br>Pamiparib 2 Weeks + RT (N = 3)	Pamiparib 4 Weeks + RT (N = 8)	Pamiparib 6 Weeks + RT (N = 9)	Arm A: Dose-expansion Phase<br>Pamiparib 6 Weeks + RT (N = 40)	All Patients (N = 60)
Agitation	0 (0.0)	0 (0.0)	0 (0.0)	5 (12.5)	5 (8.3)
Blood and lymphatic system disorders	0 (0.0)	1 (12.5)	0 (0.0)	18 (45.0)	19 (31.7)
Anemia	0 (0.0)	1 (12.5)	0 (0.0)	13 (32.5)	14 (23.3)
Thrombocytopenia	0 (0.0)	0 (0.0)	0 (0.0)	4 (10.0)	4 (6.7)
Neutropenia	0 (0.0)	0 (0.0)	0 (0.0)	3 (7.5)	3 (5.0)
Respiratory, thoracic, and mediastinal disorders	0 (0.0)	3 (37.5)	2 (22.2)	12 (30.0)	17 (28.3)
Cough	0 (0.0)	1 (12.5)	0 (0.0)	4 (10.0)	5 (8.3)
Nasal congestion	0 (0.0)	1 (12.5)	1 (11.1)	3 (7.5)	5 (8.3)
Pulmonary embolism	0 (0.0)	0 (0.0)	0 (0.0)	4 (10.0)	4 (6.7)
Infections and infestations	0 (0.0)	2 (25.0)	0 (0.0)	14 (35.0)	16 (26.7)
Otitis media	0 (0.0)	1 (12.5)	0 (0.0)	2 (5.0)	3 (5.0)
Injury, poisoning, and procedural complications	0 (0.0)	3 (37.5)	0 (0.0)	10 (25.0)	13 (21.7)
Fall	0 (0.0)	1 (12.5)	0 (0.0)	5 (12.5)	6 (10.0)
Musculoskeletal and connective tissue disorders	0 (0.0)	2 (25.0)	1 (11.1)	10 (25.0)	13 (21.7)
Back pain	0 (0.0)	0 (0.0)	0 (0.0)	3 (7.5)	3 (5.0)
Muscular weakness	0 (0.0)	1 (12.5)	1 (11.1)	1 (2.5)	3 (5.0)
Renal and urinary disorders	0 (0.0)	0 (0.0)	1 (11.1)	12 (30.0)	13 (21.7)
Urinary incontinence	0 (0.0)	0 (0.0)	0 (0.0)	6 (15.0)	6 (10.0)
Hematuria	0 (0.0)	0 (0.0)	1 (11.1)	2 (5.0)	3 (5.0)
Ear and labyrinth disorders	0 (0.0)	2 (25.0)	2 (22.2)	7 (17.5)	11 (18.3)
Hypoacusis	0 (0.0)	0 (0.0)	1 (11.1)	2 (5.0)	3 (5.0)
Tinnitus	0 (0.0)	0 (0.0)	0 (0.0)	3 (7.5)	3 (5.0)
Eye disorders	0 (0.0)	1 (12.5)	1 (11.1)	6 (15.0)	8 (13.3)
Vision blurred	0 (0.0)	1 (12.5)	0 (0.0)	3 (7.5)	4 (6.7)

Data cutoff: 13 April 2021.
Note: Patients with multiple events for a given Preferred Term and System Organ Class are counted only once for the Preferred Term and System Organ Class, respectively. Events are sorted by decreasing frequency of System Organ Class and Preferred Term in the “all patients” column. AEs are coded using the MedDRA version 23.0.
Abbreviations: AE, adverse event; MedDRA, Medical Dictionary for Regulatory Activities; RT, radiation therapy; TEAE, treatment-emergent adverse event.

Supplementary Table S6. Newly-diagnosed glioblastoma (Arm B): TEAEs by system organ class and preferred term in two or more patients (safety analysis set).

	Dose-escalation Phase
**System Organ Class**<br>**Preferred Term, N (%)**	**Pamiparib 6 Weeks + RT + TMZ 60 mg Weeks 1 and 5 (N = 9)**
Patients with ≥1 TEAE	9 (100.0)
Gastrointestinal disorders	8 (88.9)
Nausea	7 (77.8)
Constipation	3 (33.3)
Diarrhea	2 (22.2)
Vomiting	2 (22.2)
General disorders and administration site conditions	6 (66.7)
Fatigue	6 (66.7)
Gait disturbance	2 (22.2)
Edema peripheral	2 (22.2)
Investigations	6 (66.7)
White blood cell count decreased	4 (44.4)
Neutrophil count decreased	3 (33.3)
Lymphocyte count decreased	2 (22.2)
Metabolism and nutrition disorders	6 (66.7)
Decreased appetite	4 (44.4)
Hypokalemia	2 (22.2)
Nervous system disorders	6 (66.7)
Hemiparesis	3 (33.3)
Dizziness	2 (22.2)
Dysgeusia	2 (22.2)
Headache	2 (22.2)
Skin and subcutaneous tissue disorders	6 (66.7)
Alopecia	4 (44.4)
Rash maculo-papular	3 (33.3)
Blood and lymphatic system disorders	5 (55.6)
Anemia	4 (44.4)
Infections and infestations	4 (44.4)
Oral candidiasis	2 (22.2)
Musculoskeletal and connective tissue disorders	4 (44.4)
Arthralgia	2 (22.2)
Psychiatric disorders	4 (44.4)
Anxiety	2 (22.2)
Insomnia	2 (22.2)
Renal and urinary disorders	4 (44.4)
Urinary incontinence	2 (22.2)
Vascular disorders	4 (44.4)
Hypertension	2 (22.2)

Data cutoff: 13 April 2021.

Patients with multiple events for a given Preferred Term and System Organ Class are counted only once for the Preferred Term and System Organ Class, respectively. Events are sorted by decreasing

frequency of System Organ Class and Preferred Term. AEs are coded using the MedDRA version 23.0.

Abbreviations: AE, adverse event; MedDRA, Medical Dictionary for Regulatory Activities; RT, radiation therapy; TEAE, treatment-emergent adverse event; TMZ, temozolomide.

Supplementary Table S7. Recurrent/refractory glioblastoma (Arm C): TEAEs by system organ class and preferred term in ≥5% of patients (in "all patients" column) (safety analysis set).

System Organ Class<br>Preferred Term, N (%)	Dose-escalation Phase<br>Pamiparib + TMZ 20 mg<br>Days 1–21 (N = 9)	Dose-escalation Phase<br>Pamiparib + TMZ 40 mg<br>Days 1–21 (N = 8)	Dose-expansion Phase<br>Pamiparib + TMZ 60 mg<br>Days 1–7 (N = 30)	All Patients<br>(N = 47)
Patients with ≥1 TEAE	9 (100.0)	8 (100.0)	29 (96.7)	46 (97.9)
Gastrointestinal disorders	6 (66.7)	7 (87.5)	20 (66.7)	33 (70.2)
Nausea	4 (44.4)	5 (62.5)	13 (43.3)	22 (46.8)
Constipation	2 (22.2)	2 (25.0)	12 (40.0)	16 (34.0)
Vomiting	1 (11.1)	5 (62.5)	7 (23.3)	13 (27.7)
Diarrhea	2 (22.2)	1 (12.5)	4 (13.3)	7 (14.9)
General disorders and administration site conditions	6 (66.7)	5 (62.5)	21 (70.0)	32 (68.1)
Fatigue	1 (11.1)	5 (62.5)	17 (56.7)	23 (48.9)
Gait disturbance	1 (11.1)	1 (12.5)	3 (10.0)	5 (10.6)
Edema peripheral	1 (11.1)	0 (0.0)	3 (10.0)	4 (8.5)
Pyrexia	1 (11.1)	0 (0.0)	3 (10.0)	4 (8.5)
Nervous system disorders	5 (55.6)	3 (37.5)	23 (76.7)	31 (66.0)
Dizziness	1 (11.1)	1 (12.5)	8 (26.7)	10 (21.3)
Headache	1 (11.1)	0 (0.0)	8 (26.7)	9 (19.1)
Hemiparesis	4 (44.4)	1 (12.5)	4 (13.3)	9 (19.1)
Seizure	2 (22.2)	0 (0.0)	4 (13.3)	6 (12.8)
Aphasia	1 (11.1)	0 (0.0)	4 (13.3)	5 (10.6)
Dysgeusia	1 (11.1)	0 (0.0)	2 (6.7)	3 (6.4)
Paresthesia	1 (11.1)	0 (0.0)	2 (6.7)	3 (6.4)
Investigations	5 (55.6)	3 (37.5)	11 (36.7)	19 (40.4)
Platelet count decreased	3 (33.3)	1 (12.5)	8 (26.7)	12 (25.5)
Neutrophil count decreased	2 (22.2)	2 (25.0)	5 (16.7)	9 (19.1)
White blood cell count decreased	2 (22.2)	1 (12.5)	6 (20.0)	9 (19.1)
Lymphocyte count decreased	3 (33.3)	1 (12.5)	4 (13.3)	8 (17.0)
Blood bilirubin increased	2 (22.2)	1 (12.5)	1 (3.3)	4 (8.5)
Weight decreased	0 (0.0)	1 (12.5)	3 (10.0)	4 (8.5)

System Organ Class<br>Preferred Term, N (%)	Dose-escalation Phase<br>Pamiparib + TMZ 20 mg<br>Days 1–21 (N = 9)	Pamiparib + TMZ 40 mg<br>Days 1–21 (N = 8)	Dose-expansion Phase<br>Pamiparib + TMZ 60 mg<br>Days 1–7 (N = 30)	All Patients<br>(N = 47)
Blood creatinine increased	0 (0.0)	1 (12.5)	2 (6.7)	3 (6.4)
Metabolism and nutrition disorders	4 (44.4)	3 (37.5)	10 (33.3)	17 (36.2)
Decreased appetite	2 (22.2)	1 (12.5)	7 (23.3)	10 (21.3)
Hyperglycemia	2 (22.2)	0 (0.0)	1 (3.3)	3 (6.4)
Blood and lymphatic system disorders	3 (33.3)	3 (37.5)	10 (33.3)	16 (34.0)
Anemia	3 (33.3)	3 (37.5)	6 (20.0)	12 (25.5)
Lymphopenia	0 (0.0)	0 (0.0)	4 (13.3)	4 (8.5)
Neutropenia	0 (0.0)	1 (12.5)	3 (10.0)	4 (8.5)
Thrombocytopenia	0 (0.0)	1 (12.5)	3 (10.0)	4 (8.5)
Musculoskeletal and connective tissue disorders	3 (33.3)	1 (12.5)	12 (40.0)	16 (34.0)
Muscular weakness	2 (22.2)	1 (12.5)	5 (16.7)	8 (17.0)
Myalgia	0 (0.0)	0 (0.0)	3 (10.0)	3 (6.4)
Psychiatric disorders	1 (11.1)	2 (25.0)	10 (33.3)	13 (27.7)
Confusional state	0 (0.0)	1 (12.5)	4 (13.3)	5 (10.6)
Insomnia	0 (0.0)	1 (12.5)	4 (13.3)	5 (10.6)
Depression	0 (0.0)	0 (0.0)	3 (10.0)	3 (6.4)
Respiratory, thoracic, and mediastinal disorders	1 (11.1)	2 (25.0)	10 (33.3)	13 (27.7)
Dyspnea	0 (0.0)	0 (0.0)	4 (13.3)	4 (8.5)
Nasal congestion	0 (0.0)	1 (12.5)	3 (10.0)	4 (8.5)
Cough	1 (11.1)	0 (0.0)	2 (6.7)	3 (6.4)
Injury, poisoning, and procedural complications	0 (0.0)	4 (50.0)	7 (23.3)	11 (23.4)
Fall	0 (0.0)	3 (37.5)	6 (20.0)	9 (19.1)
Contusion	0 (0.0)	0 (0.0)	3 (10.0)	3 (6.4)
Skin and subcutaneous tissue disorders	2 (22.2)	0 (0.0)	5 (16.7)	7 (14.9)
Rash maculo-papular	2 (22.2)	0 (0.0)	2 (6.7)	4 (8.5)
Vascular disorders	1 (11.1)	1 (12.5)	5 (16.7)	7 (14.9)
Hypotension	1 (11.1)	0 (0.0)	2 (6.7)	3 (6.4)

Data cutoff: 13 April 2021.

Note: Patients with multiple events for a given Preferred Term and System Organ Class are counted only once for the Preferred Term and System Organ Class, respectively. Events are sorted by decreasing frequency of System Organ Class and Preferred Term in the "all patients" column. AEs are coded using the MedDRA version 23.0.
Abbreviations: AE, adverse event; MedDRA, Medical Dictionary for Regulatory Activities; TEAE, treatment-emergent adverse event; TMZ, temozolomide.

Supplementary Table S8. Newly-diagnosed glioblastoma (Arm A): grade ≥ 3 TEAEs by system organ class and preferred term (safety analysis set).

System Organ Class<br>Preferred Term, N (%)	Arm A: Dose-escalation Phase<br>Pamiparib 2 Weeks + RT<br>(N = 3)	Arm A: Dose-escalation Phase<br>Pamiparib 4 Weeks + RT<br>(N = 8)	Arm A: Dose-escalation Phase<br>Pamiparib 6 Weeks + RT<br>(N = 9)	Arm A: Dose-expansion Phase<br>Pamiparib 6 Weeks + RT<br>(N = 40)	All Patients<br>(N = 60)
Patients with ≥1 TEAE	1 (33.3)	3 (37.5)	4 (44.4)	25 (62.5)	33 (55.0)
Nervous system disorders	0 (0.0)	1 (12.5)	1 (11.1)	11 (27.5)	13 (21.7)
Seizure	0 (0.0)	0 (0.0)	0 (0.0)	2 (5.0)	2 (3.3)
Vasogenic cerebral edema	0 (0.0)	1 (12.5)	0 (0.0)	1 (2.5)	2 (3.3)
Apraxia	0 (0.0)	0 (0.0)	0 (0.0)	1 (2.5)	1 (1.7)
Brain edema	0 (0.0)	0 (0.0)	0 (0.0)	1 (2.5)	1 (1.7)
Cerebral cyst	0 (0.0)	0 (0.0)	0 (0.0)	1 (2.5)	1 (1.7)
Headache	0 (0.0)	0 (0.0)	0 (0.0)	1 (2.5)	1 (1.7)
Hemiplegia	0 (0.0)	0 (0.0)	0 (0.0)	1 (2.5)	1 (1.7)
Hydrocephalus	0 (0.0)	0 (0.0)	0 (0.0)	1 (2.5)	1 (1.7)
Partial seizures	0 (0.0)	0 (0.0)	0 (0.0)	1 (2.5)	1 (1.7)
Peripheral motor neuropathy	0 (0.0)	0 (0.0)	1 (11.1)	0 (0.0)	1 (1.7)
Psychogenic seizure	0 (0.0)	0 (0.0)	0 (0.0)	1 (2.5)	1 (1.7)
Syncope	0 (0.0)	0 (0.0)	0 (0.0)	1 (2.5)	1 (1.7)
Blood and lymphatic system disorders	0 (0.0)	1 (12.5)	0 (0.0)	7 (17.5)	8 (13.3)
Anemia	0 (0.0)	1 (12.5)	0 (0.0)	5 (12.5)	6 (10.0)
Neutropenia	0 (0.0)	0 (0.0)	0 (0.0)	2 (5.0)	2 (3.3)
Lymphopenia	0 (0.0)	0 (0.0)	0 (0.0)	1 (2.5)	1 (1.7)
General disorders and administration site conditions	0 (0.0)	0 (0.0)	3 (33.3)	3 (7.5)	6 (10.0)
Fatigue	0 (0.0)	0 (0.0)	1 (11.1)	2 (5.0)	3 (5.0)
Asthenia	0 (0.0)	0 (0.0)	1 (11.1)	0 (0.0)	1 (1.7)
Chills	0 (0.0)	0 (0.0)	1 (11.1)	0 (0.0)	1 (1.7)
Gait disturbance	0 (0.0)	0 (0.0)	0 (0.0)	1 (2.5)	1 (1.7)
Investigations	0 (0.0)	0 (0.0)	0 (0.0)	6 (15.0)	6 (10.0)

System Organ Class<br>Preferred Term, N (%)	Arm A: Dose-escalation Phase<br>Pamiparib 2 Weeks + RT<br>(N = 3)	Arm A: Dose-escalation Phase<br>Pamiparib 4 Weeks + RT<br>(N = 8)	Arm A: Dose-escalation Phase<br>Pamiparib 6 Weeks + RT<br>(N = 9)	Arm A: Dose-expansion Phase<br>Pamiparib 6 Weeks + RT<br>(N = 40)	All Patients<br>(N = 60)
Alanine aminotransferase increased	0 (0.0)	0 (0.0)	0 (0.0)	2 (5.0)	2 (3.3)
Neutrophil count decreased	0 (0.0)	0 (0.0)	0 (0.0)	2 (5.0)	2 (3.3)
White blood cell count decreased	0 (0.0)	0 (0.0)	0 (0.0)	2 (5.0)	2 (3.3)
Lymphocyte count decreased	0 (0.0)	0 (0.0)	0 (0.0)	1 (2.5)	1 (1.7)
Gastrointestinal disorders	0 (0.0)	2 (25.0)	1 (11.1)	2 (5.0)	5 (8.3)
Nausea	0 (0.0)	0 (0.0)	1 (11.1)	1 (2.5)	2 (3.3)
Abdominal pain	0 (0.0)	0 (0.0)	0 (0.0)	1 (2.5)	1 (1.7)
Constipation	0 (0.0)	0 (0.0)	0 (0.0)	1 (2.5)	1 (1.7)
Diarrhea	0 (0.0)	1 (12.5)	0 (0.0)	0 (0.0)	1 (1.7)
Gastrointestinal hemorrhage	0 (0.0)	1 (12.5)	0 (0.0)	0 (0.0)	1 (1.7)
Proctalgia	0 (0.0)	0 (0.0)	0 (0.0)	1 (2.5)	1 (1.7)
Vomiting	0 (0.0)	0 (0.0)	0 (0.0)	1 (2.5)	1 (1.7)
Metabolism and nutrition disorders	1 (33.3)	0 (0.0)	1 (11.1)	2 (5.0)	4 (6.7)
Dehydration	0 (0.0)	0 (0.0)	0 (0.0)	1 (2.5)	1 (1.7)
Hyperglycemia	1 (33.3)	0 (0.0)	0 (0.0)	0 (0.0)	1 (1.7)
Hypokalemia	0 (0.0)	0 (0.0)	1 (11.1)	0 (0.0)	1 (1.7)
Hyponatremia	0 (0.0)	0 (0.0)	0 (0.0)	1 (2.5)	1 (1.7)
Respiratory, thoracic, and mediastinal disorders	0 (0.0)	0 (0.0)	0 (0.0)	4 (10.0)	4 (6.7)
Pulmonary embolism	0 (0.0)	0 (0.0)	0 (0.0)	4 (10.0)	4 (6.7)
Infections and infestations	0 (0.0)	1 (12.5)	0 (0.0)	2 (5.0)	3 (5.0)
Bronchitis	0 (0.0)	0 (0.0)	0 (0.0)	1 (2.5)	1 (1.7)
Sepsis	0 (0.0)	0 (0.0)	0 (0.0)	1 (2.5)	1 (1.7)
Urinary tract infection	0 (0.0)	1 (12.5)	0 (0.0)	0 (0.0)	1 (1.7)
Wound infection	0 (0.0)	0 (0.0)	0 (0.0)	1 (2.5)	1 (1.7)

System Organ Class<br>Preferred Term, N (%)	Arm A: Dose-escalation Phase<br>Pamiparib 2 Weeks + RT<br>(N = 3)	Pamiparib 4 Weeks + RT<br>(N = 8)	Pamiparib 6 Weeks + RT<br>(N = 9)	Arm A: Dose-expansion Phase<br>Pamiparib 6 Weeks + RT<br>(N = 40)	All Patients<br>(N = 60)
Ear and labyrinth disorders	0 (0.0)	0 (0.0)	1 (11.1)	1 (2.5)	2 (3.3)
Hypoacusis	0 (0.0)	0 (0.0)	0 (0.0)	1 (2.5)	1 (1.7)
Vertigo	0 (0.0)	0 (0.0)	1 (11.1)	0 (0.0)	1 (1.7)
Psychiatric disorders	0 (0.0)	0 (0.0)	0 (0.0)	2 (5.0)	2 (3.3)
Agitation	0 (0.0)	0 (0.0)	0 (0.0)	1 (2.5)	1 (1.7)
Mental status changes	0 (0.0)	0 (0.0)	0 (0.0)	1 (2.5)	1 (1.7)
Suicide attempt	0 (0.0)	0 (0.0)	0 (0.0)	1 (2.5)	1 (1.7)
Renal and urinary disorders	0 (0.0)	0 (0.0)	0 (0.0)	1 (2.5)	1 (1.7)
Nephrolithiasis	0 (0.0)	0 (0.0)	0 (0.0)	1 (2.5)	1 (1.7)
Skin and subcutaneous tissue disorders	0 (0.0)	0 (0.0)	0 (0.0)	1 (2.5)	1 (1.7)
Rash	0 (0.0)	0 (0.0)	0 (0.0)	1 (2.5)	1 (1.7)
Vascular disorders	0 (0.0)	0 (0.0)	0 (0.0)	1 (2.5)	1 (1.7)
Hypertension	0 (0.0)	0 (0.0)	0 (0.0)	1 (2.5)	1 (1.7)

Data cutoff: 13 April 2021.

Note: Patients with multiple events for a given Preferred Term and System Organ Class are counted only once for the Preferred Term and System Organ Class, respectively. Events are sorted by decreasing frequency of System Organ Class and Preferred Term in the “all patients” column. AEs are coded using the MedDRA version 23.0 and are graded according to the CTCAE version 4.03.

Abbreviations: AE, adverse event; CTCAE, Common Terminology Criteria for Adverse Events; MedDRA, Medical Dictionary For Regulatory Activities; RT, radiation therapy; TEAE, treatment-emergent adverse event.

Supplementary Table S9. Newly-diagnosed glioblastoma (Arm B): grade ≥ 3 TEAEs by system organ class and preferred term (safety analysis set).

System Organ Class<br>Preferred Term, N (%)	Dose-escalation Phase<br>Pamiparib 6 Weeks + RT + TMZ 60 mg Weeks 1 and 5<br>(N = 9)
Patients with ≥1 TEAE	4 (44.4)
Blood and lymphatic system disorders	3 (33.3)
Anemia	1 (11.1)
Febrile neutropenia	1 (11.1)
Leukopenia	1 (11.1)
Neutropenia	1 (11.1)
Investigations	3 (33.3)
Neutrophil count decreased	2 (22.2)
White blood cell count decreased	2 (22.2)
Lymphocyte count decreased	1 (11.1)
Nervous system disorders	2 (22.2)
Depressed level of consciousness	1 (11.1)
Headache	1 (11.1)
Seizure	1 (11.1)
General disorders and administration site conditions	1 (11.1)
Fatigue	1 (11.1)
Neoplasms benign, malignant, and unspecified (including cysts and polyps)	1 (11.1)
Tumor flare	1 (11.1)
Vascular disorders	1 (11.1)
Hypertension	1 (11.1)

Data cutoff: 13 April 2021.
Note: Patients with multiple events for a given Preferred Term and System Organ Class are counted only once for the Preferred Term and System Organ Class, respectively. Events are sorted by decreasing frequency of System Organ Class and Preferred Term. AEs are coded using the MedDRA version 23.0 and are graded according to the CTCAE version 4.03.
Abbreviations: AE, adverse event; CTCAE, Common Terminology Criteria for Adverse Events; MedDRA, Medical Dictionary for Regulatory Activities; RT, radiation therapy; TEAE, treatment-emergent adverse event; TMZ, temozolomide.

Supplementary Table S10. Recurrent/refractory glioblastoma (Arm C): grade ≥ 3 TEAEs by system organ class and preferred term (safety analysis set).

System Organ Class<br>Preferred Term, N (%)	Dose-escalation phase<br>Pamiparib + TMZ 20 mg<br>Days 1–21 (N = 9)	Pamiparib + TMZ 40 mg<br>Days 1–21 (N = 8)	Dose-expansion phase<br>Pamiparib + TMZ 60 mg<br>Days 1–7 (N = 30)	All patients<br>(N = 47)
Patients with ≥1 TEAE	5 (55.6)	7 (87.5)	19 (63.3)	31 (66.0)
Investigations	2 (22.2)	2 (25.0)	8 (26.7)	12 (25.5)
Lymphocyte count decreased	1 (11.1)	1 (12.5)	3 (10.0)	5 (10.6)
Neutrophil count decreased	0 (0.0)	2 (25.0)	2 (6.7)	4 (8.5)
Platelet count decreased	0 (0.0)	1 (12.5)	3 (10.0)	4 (8.5)
White blood cell count decreased	0 (0.0)	1 (12.5)	3 (10.0)	4 (8.5)
Alanine aminotransferase increased	1 (11.1)	0 (0.0)	0 (0.0)	1 (2.1)
Aspartate aminotransferase increased	1 (11.1)	0 (0.0)	0 (0.0)	1 (2.1)
Blood and lymphatic system disorders	1 (11.1)	3 (37.5)	6 (20.0)	10 (21.3)
Anemia	1 (11.1)	3 (37.5)	1 (3.3)	5 (10.6)
Neutropenia	0 (0.0)	1 (12.5)	3 (10.0)	4 (8.5)
Thrombocytopenia	0 (0.0)	1 (12.5)	2 (6.7)	3 (6.4)
Lymphopenia	0 (0.0)	0 (0.0)	2 (6.7)	2 (4.3)
Leukopenia	0 (0.0)	0 (0.0)	1 (3.3)	1 (2.1)
General disorders and administration site conditions	1 (11.1)	2 (25.0)	3 (10.0)	6 (12.8)
Fatigue	1 (11.1)	2 (25.0)	2 (6.7)	5 (10.6)
Impaired healing	0 (0.0)	0 (0.0)	1 (3.3)	1 (2.1)
Nervous system disorders	2 (22.2)	1 (12.5)	3 (10.0)	6 (12.8)
Headache	0 (0.0)	0 (0.0)	2 (6.7)	2 (4.3)
Hemiparesis	2 (22.2)	0 (0.0)	0 (0.0)	2 (4.3)
Cerebrovascular accident	1 (11.1)	0 (0.0)	0 (0.0)	1 (2.1)
Encephalopathy	1 (11.1)	0 (0.0)	0 (0.0)	1 (2.1)
Partial seizures	0 (0.0)	0 (0.0)	1 (3.3)	1 (2.1)
Vasogenic cerebral edema	0 (0.0)	1 (12.5)	0 (0.0)	1 (2.1)
Psychiatric disorders	0 (0.0)	1 (12.5)	3 (10.0)	4 (8.5)

System Organ Class<br>Preferred Term, N (%)	Dose-escalation phase<br>Pamiparib + TMZ 20 mg<br>Days 1–21 (N = 9)	Pamiparib + TMZ 40 mg<br>Days 1–21 (N = 8)	Dose-expansion phase<br>Pamiparib + TMZ 60 mg<br>Days 1–7 (N = 30)	All patients<br>(N = 47)
Confusional state	0 (0.0)	1 (12.5)	2 (6.7)	3 (6.4)
Depression	0 (0.0)	0 (0.0)	1 (3.3)	1 (2.1)
Respiratory, thoracic, and mediastinal disorders	1 (11.1)	1 (12.5)	2 (6.7)	4 (8.5)
Pulmonary embolism	0 (0.0)	1 (12.5)	1 (3.3)	2 (4.3)
Hypoxia	1 (11.1)	0 (0.0)	0 (0.0)	1 (2.1)
Laryngeal hemorrhage	0 (0.0)	0 (0.0)	1 (3.3)	1 (2.1)
Vascular disorders	1 (11.1)	0 (0.0)	3 (10.0)	4 (8.5)
Embolism	0 (0.0)	0 (0.0)	2 (6.7)	2 (4.3)
Hematoma	1 (11.1)	0 (0.0)	0 (0.0)	1 (2.1)
Hypotension	0 (0.0)	0 (0.0)	1 (3.3)	1 (2.1)
Gastrointestinal disorders	0 (0.0)	1 (12.5)	2 (6.7)	3 (6.4)
Nausea	0 (0.0)	1 (12.5)	2 (6.7)	3 (6.4)
Vomiting	0 (0.0)	1 (12.5)	0 (0.0)	1 (2.1)
Musculoskeletal and connective tissue disorders	1 (11.1)	0 (0.0)	2 (6.7)	3 (6.4)
Muscular weakness	1 (11.1)	0 (0.0)	2 (6.7)	3 (6.4)
Infections and infestations	0 (0.0)	0 (0.0)	2 (6.7)	2 (4.3)
Pneumonia	0 (0.0)	0 (0.0)	1 (3.3)	1 (2.1)
Wound infection	0 (0.0)	0 (0.0)	1 (3.3)	1 (2.1)
Injury, poisoning, and procedural complications	0 (0.0)	0 (0.0)	2 (6.7)	2 (4.3)
Fall	0 (0.0)	0 (0.0)	2 (6.7)	2 (4.3)
Neoplasms benign, malignant, and unspecified (including cysts and polyps)	0 (0.0)	0 (0.0)	2 (6.7)	2 (4.3)
Malignant melanoma	0 (0.0)	0 (0.0)	1 (3.3)	1 (2.1)
Tumor flare	0 (0.0)	0 (0.0)	1 (3.3)	1 (2.1)
Cardiac disorders	0 (0.0)	0 (0.0)	1 (3.3)	1 (2.1)
Atrial fibrillation	0 (0.0)	0 (0.0)	1 (3.3)	1 (2.1)
Renal and urinary disorders	0 (0.0)	0 (0.0)	1 (3.3)	1 (2.1)

System Organ Class<br>Preferred Term, N (%)	Dose-escalation phase<br>Pamiparib + TMZ 20 mg<br>Days 1–21 (N = 9)	 Pamiparib + TMZ 40 mg<br>Days 1–21 (N = 8)	Dose-expansion phase<br>Pamiparib + TMZ 60 mg<br>Days 1–7 (N = 30)	All patients<br>(N = 47)
Nephrolithiasis	0 (0.0)	0 (0.0)	1 (3.3)	1 (2.1)

Data cutoff: 13 April 2021.
Note: Patients with multiple events for a given Preferred Term and System Organ Class are counted only once for the Preferred Term and System Organ Class, respectively. Events are sorted by decreasing frequency of System Organ Class and Preferred Term in the "all patients" column. AEs are coded using the MedDRA version 23.0 and are graded according to the CTCAE version 4.03.
Abbreviations: AE, adverse event; CTCAE, Common Terminology Criteria for Adverse Events; MedDRA, Medical Dictionary for Regulatory Activities; TEAE, treatment-emergent adverse event; TMZ, temozolomide.
